# Developing a dynamic combined power quality index for assessing the performance of a nuclear facility

**DOI:** 10.1038/s41598-025-89383-5

**Published:** 2025-02-25

**Authors:** Asmaa M. Elsotohy, Mohammed Hamouda Ali, Ahmed S. Adail, Ayman A. Eisa, El-said A Othman

**Affiliations:** 1 Department of Nuclear Safety and Radiological Emergencies, NCRRT, Egyptian Atomic Energy Authority, Cairo, Egypt; 2 Department of Electrical Engineering, Faculty of Engineering, Al-Azhar University, Cairo, Egypt; 3 Department of Fuel Technology, Hot Laboratory Centre, Egyptian Atomic Energy Authority, Cairo, Egypt

**Keywords:** Power quality, MCDM, Nuclear research reactor, Compound power quality index, Engineering, Mathematics and computing, Nuclear energy

## Abstract

Studying and evaluating the power quality (PQ) of an electrical network for nuclear installation is an important issue and a hot research topic for guaranteeing reliable and safe operation of sensitive electrical loads during this type of installation. As several PQ phenomena determine the overall PQ performance, analyzing PQ signals for evaluating the overall PQ is one of the major challenges for researchers in this field. Technically, voltage imbalance, current imbalance, voltage harmonic distortion, current harmonic distortion, and the power factor are five important PQ phenomena that judge the overall PQ performance of an electrical system. Multicriteria decision-making (MCDM) is used here as a methodology to identify a weighting for each PQ phenomenon. This paper proposes a power quality evaluation method for a nuclear research reactor (NRR) electrical network based on two MCDM. Methods the analytic hierarchy process (AHP) and criterion importance through inter-criteria correlation (CRITIC). A MATLAB/Simulink model for the NRR electrical system is presented, and then its validity and credibility are verified via measurements. In this study, different abnormal conditions are simulated in the NRR network to generate power quality disturbances, including a three-phase nonlinear load to simulate harmonics, an unbalanced load to simulate unbalance, and an inductive load to simulate the change in the power factor. The effectiveness and robustness of the proposed methodology are demonstrated through these different case studies. The results show that the obtained CPQI based on the dynamic weight approach allows for more accurate evaluations by adjusting the importance of various PQ phenomena depending on operational conditions and priorities. The main contribution of this paper is that a single compound power quality index (CPQI) was developed based on both the dynamic weights obtained from AHP-CRITIC methods and the results of the five PQ phenomena obtained under different abnormal conditions, considering the threshold level for each of these PQ phenomena. The analysis of the obtained results shows that this method accurately evaluates the overall PQ performance.

## Introduction

The power quality deals with current and voltage waveform deviations from their ideal behaviors in the electrical power system, from generation to consumption. These deviations in the waveforms of current or voltage may be called power quality disturbances^1^. The importance of power quality is because a high-quality electrical source can deliver all the electrical energy needed without any change in voltage. In previous decades, most power quality problems were those that affected power distribution, such as lightning, line or transformer failures, and/or very high electrical demands (brownouts) on the electrical network. However, currently, most power quality problems are due to technological changes and the way electricity is now being used by people^2^.

IEEE 1159–2019 presents in detail the categories and descriptions of the different electromagnetic PQ phenomena that can cause power quality problems^3^. Nonlinear loads, such as those applied in electronic devices, are considered to cause pollution to the PQ of an electrical network^4^. On the other hand, connecting unbalanced loads to the electrical network may lead to voltage and current imbalances^5^. The electrical PQ is considered one of the most important factors for ensuring the continuous and reliable operation of sensitive devices and equipment in nuclear facilities^6^. Assessing the power quality is influenced by renewable energy systems. Studies have shown that the variability and intermittency of renewable energy sources can challenge voltage stability^7,8^. The integration of electric vehicles further complicates these challenges, as the increasing demand for charging stations and vehicle-to-grid interactions can exacerbate grid imbalances and increase power losses^9,10^. Many researchers are concerned with studying the effect of inserting power quality improvement techniques to enhance the voltage stability^11,12^. Others focused on selecting the optimal size and location of renewable energy sources from the view of power losses and cost^13–15^. With the world’s trend towards the importance of green energy and concerns with reducing CO2 emissions, many researchers began taking into account the environmental impact in selecting the type and location of renewable energy resources^16,17^. Moreover, the optimal design of renewable energy sources with the aim of reducing the total harmonic distortion is discussed in^18,19^with different algorithms. Also, others are concerned with mitigating problems in different PQ phenomena using PQ improvement techniques of an electrical network connected to renewable energy sources^20,21^.

To reach the optimal solution in the presence of many similar options, a scientific, strategic, and efficient evaluation method must be adopted to determine the PQ using a single indicator. To optimize the selection process, a basis is needed that can help rank the given alternatives. To do so, MCDM can be used because it provides the composition of a number of alternatives and multiple criteria and compares them in some manner^22^. Decision-making is a selective decision process in which relevant information is gathered and organized, and alternatives are identified and evaluated^23^. The weighting of multiple criteria based on MCDM may depend on the decision maker’s opinions, which are called subjective weights; others depend on solving mathematical models without considering the decision maker’s opinions, which are called objective weights^24^. MCDM can be applied in diverse applications across various domains, providing decision-makers with appreciated support in complex decision problems^25,26^. In management and business, MCDM techniques have been utilized for project selection, strategic planning, investment decisions, and supplier evaluation. In technology and engineering, these methods have supported process optimization, product design, and technology selection^27^. Additionally, MCDM methods are applied for evaluating environmental impact, sustainability assessment, and resource allocation^28,29^. Several studies have addressed the applications of MCDM in different energy-related fields. Some focus on energy policymaking, and others are on the site selection of wind farms, solar photovoltaics, and hydropower plants^30^. Moreover, several articles have focused on studying and evaluating PQ performance based on MCDM.

Several published articles have investigated and evaluated PQ performance using a number of different methodologies. However, several of these studies focused on using subjective MCDM methods^31–34^, and^35^, while others focused on evaluating the PQ phenomenon based only on objective MCDM^36,37^. Some of the recent related research has focused on combining subjective and objective MCDM methods. In^38^, Yuan Xiangyu and others evaluated the performance of smart meters based on AHP-CRITIC. This method combines expert opinion with an objective index and overcomes the shortcomings of traditional methods, which depend only on expert experience. In^39^, the AHP-CRITIC dynamic weight method was used to assess equipment health status. In^40^, the AHP-entropy weight (EW) combination weighting method was used to evaluate the PQ of an electrical grid in China, and then an improved technique for order preference by similarity to the ideal solution (TOPSIS) method was used to rank the different samples. In^41^, the AHP was combined with an improved CRITIC method in a fuzzy environment to evaluate the PQ of a distribution network. However, this study did not consider the standard level of each PQ phenomenon when evaluating the overall power quality and depended only on the static weight. An overview of the contributions and gaps in the PQ assessment literature can be found in Table 1.

A PQ assessment of the electrical system of a nuclear installation using AHP-CRITIC hybrid methods is presented in the present study. First, the AHP method calculates the subjective weight of each PQ phenomenon; then, based on measurements and using the CRITIC method, the objective weights are calculated. By combining the two weights, a comprehensive weight is obtained. The proposed approach is verified on the NRR electrical system in MATLAB/Simulink. Additionally, the combined PQ index is calculated by taking into consideration the standard level of each PQ phenomenon and the dynamism of the weight with the change in the operation conditions of the NRR. This study mainly aimed to assess the combined PQ index of the NRR. This work can be summarized as follows:

**Table 1:** Summary of the literary contributions and gaps.

Ref.	Method	Application	Disturbances (Criteria)	Demerits	Year
^31^	AHP-based novel approach.	The proposed approach is applied in test distribution system of grid-integrated systems of renewable energy in MATLAB/Simulink.	Voltage unbalance, current and harmonic distortion, voltage harmonic distortion, voltage fluctuations, and frequency fluctuations are monitored at grid, load, and DG buses.	Using static subjective weight only.	2020
^32^	AHP	The proposed approach is applied to IEEE 13 bus test distribution system and modified by integrating the nonlinear loads and DG systems based on wind, PV and fuel cell through MATLAB/Simulink.	Voltage sags, voltage harmonics, steady-state voltage profile and voltage unbalance.	Using static subjective weight only.	2020
^33^	AHP	The proposed approach is applied to IEEE two-bus distribution network with a high degree of harmonic emissions caused by nonlinearity from solar energy conversion system on both the load and utility sides. Global Power Quality Index is identified for load, utility, and DG buses, in addition to the overall system.	Current harmonic distortion, Voltage harmonic distortion, voltage unbalance, voltage fluctuations, steady-state voltage profile, frequency fluctuations, and power factor.	Using static subjective weight only.	2021
^34^	AHP and S-transform	Distribution power network with photovoltaic, wind, and battery.	Voltage harmonic distortion, voltage deviation score, and power factor.	Using subjective weight only.	2024
^35^	AHP	A modified CIGRE low voltage microgrid network.	voltage level, current harmonic quality, voltage harmonic quality, power factor, and system frequency	Using subjective weight only.	2020
^36^	Dynamic CRITIC method	Microgrid simulation platform, which consists of wind, energy storage battery, and PV.	Harmonic, voltage deviation, three-phase unbalance, and frequency deviation.	Using dynamic objective weight only.	2021
^37^	Grey Clustering with Entropy Weight	Readings are taken from the most important buses of distributed generation systems which are: Wind bus, Photo Voltaic bus, Fuel Cell bus, Load bus, and Grid bus.	Harmonic distortion rate in current, harmonic distortion rate in voltage, voltage sag, flickers, frequency deviation, and the power factor.	Using static objective weight only.	2018
^38^	AHP- CRITIC	Evaluation of performance for three types of smart meters.	Basic error, Failure Rate, problem rectification rate, acceptance rate, test pass rate, and operation fault rate.	Using static subjective weight with static objective weight.	2021
^39^	CRITIC dynamic weight	Studying the suction nozzles health status of a certain type of chip mounter.	Blow on leakage, Vacuum on leakage, Vacuum on the blockage, Blow valve on delay, Blow valve off delay, and Vacuum on delay.	Using dynamic objective weight only.	2022
^40^	AHP-Entropy	PQ data is measured in five major nodes of a big wind farm in China.	Voltage total harmonic distortion, three-phase voltage unbalance, voltage deviation, voltage flicker, voltage fluctuation, and Frequency deviation.	Using static subjective weight with static objective weight.	2019
^41^	AHP, Improved CRITIC, and fuzzy mathematics	Taking measurements from a 10 kV distribution network.	Three-phase unbalance, Voltage Deviation, Total Harmonic Distortion Rate, and Short time flicker.	Using static subjective weight with static objective weight.	2020

- The measurements of different PQ phenomena were taken from a real network of an NRR, and after processing, these readings were used to compute the objective weight for each PQ phenomenon based on the CRITIC approach. In addition, the importance of each PQ phenomenon was identified based on expert’s opinions, and the subjective weight was computed using the AHP approach. Then, the objective and subjective weights are merged to compute the static combined weight.
- A MATLAB/Simulink model was implemented for the electrical system of the NRR, and verification and validation were performed by comparing the readings for different electrical parameters with real measurements.
- Measurements were taken from the NRR MATLAB/Simulink model of PQ phenomena under different abnormal conditions. Then, the scores of both the threshold (standard) limit and the measured values of each PQ phenomenon are computed. Additionally, based on both the static weight ($\:{W}_{s}$) and measurements from the model, the combined dynamic weight ($\:{W}_{d}$) of each PQ phenomenon under each abnormal condition is calculated. Finally, the values of the combined power quality index (CPQI) are introduced, and the results are analyzed under each abnormal condition. The obtained results showed the superiority of the proposed methodology for assessing PQ performance.

### Contribution and novelty

This article aims to formulate a novel compound power quality index (CPQI) for power quality assessment of NRR electrical systems. The development of the proposed CPQI is inspired by combining the weight from the AHP subjective MCDM method with the weight of the CRITIC objective MCDM method. This single index is expected to be sufficient for benchmarking the PQ performance, and this also aims at reducing the complexity of large data supervision-centered PQ monitoring of electrical networks. In case the magnitude of the computed CPQI is larger than the unity, then it indicates that the global PQ performance of the concerned bus, under attention, is unsatisfactory and above the reference threshold boundary. Correspondingly, if the magnitude of the CPQI is less than the unity, the healthier PQ performance of the concerned bus is under the reference threshold limit. To measure the compound PQ performance of the NRR network, five PQ phenomena, namely voltage unbalance, current unbalance, current harmonic distortion, voltage harmonic distortion, and the power factor, are selected at the NRR low-tension switchboard (LTS) at the left busbar. To enable proposed UPQI with the unique feature of benchmarking the network performance, threshold limits of all five performance indices are taken as per IEEE Std. 1159^3^. Further, dynamic weights for all four indices are proposed to ensure that the weight of each PQ phenomenon follows the change in operation conditions to assess the actual status of the electrical system. The NRR network is simulated in the MATLAB/Simulink environment for illustrating the application of the proposed PQ evaluation methodology and comparing the PQ performance of the network by the proposed approach under different abnormal operating conditions, including a three-phase nonlinear load to simulate harmonics, switching an unbalanced load to simulate unbalance, and adding an inductive load to simulate the change in the power factor. Also, a comparative analysis of the computed CPQI using static and dynamic weights is presented and analyzed. The results showed that the proposed new approach for computing the CPQI based on dynamic weight is a more effective methodology that can follow the change that occurs in the power quality of the electrical network. This kind of compound index would significantly facilitate the PQ evaluation of nuclear installation electrical systems. Also, it can be applied to any other electrical network. Moreover, it can empower the operators with the benefits of benchmarking and monitoring a single index instead of several indices. Additionally, it is very useful for helping stakeholders to understand how the PQ performance changes under the change in the operating condition of the facility.

The novelty of this work lies in several key innovative aspects that distinguish it from traditional approaches:

- **Nuclear Facility**: Unlike power quality indices for any electrical network that may not consider the specific requirements of nuclear facilities, this compound PQ index is proposed to address the particular electrical network characteristics and operational needs of a nuclear facility. Nuclear facilities have unique constraints regarding safety, reliability, and redundancy, so this index takes into account important factors such as life reduction, cost, and miss of operation for equipment.
- **MCDM Approach**: The combining of subjective and objective MCDM methods is more effective in evaluating an electrical network’s performance, taking into consideration both the real measurements and the experts’ opinions.
- **Dynamic Weighting System**: The incorporation of dynamic weights in the MCDM process represents a significant advancement. This dynamic weight of PQ phenomena makes the PQ assessment more reflective of the actual performance, which is crucial for critical environments like nuclear facilities where power quality directly impacts operational safety and efficiency.
- **Enhanced Decision Support for Operators**: The compound PQI facilitates improved decision-making for operators and maintenance teams. By combining several performance indices into a single composite index, it allows for better prioritization of actions, such as adding the suitable improvement technique. This leads to more efficient management of the electrical network and prevents other dependencies or problems that may lead to reactor shutdown or reactor scram.

In summary, the novelty of this developed compound power quality index lies in its dynamic and adaptive nature. Also, this is the first study that identifies the dynamic weight based on a hybrid AHP-CRITIC approach in the PQ field and uses this dynamic weight in computing the CPQI, considering the threshold limits and applying them to a nuclear installation.

### Paper organization

**Fig. 1:**
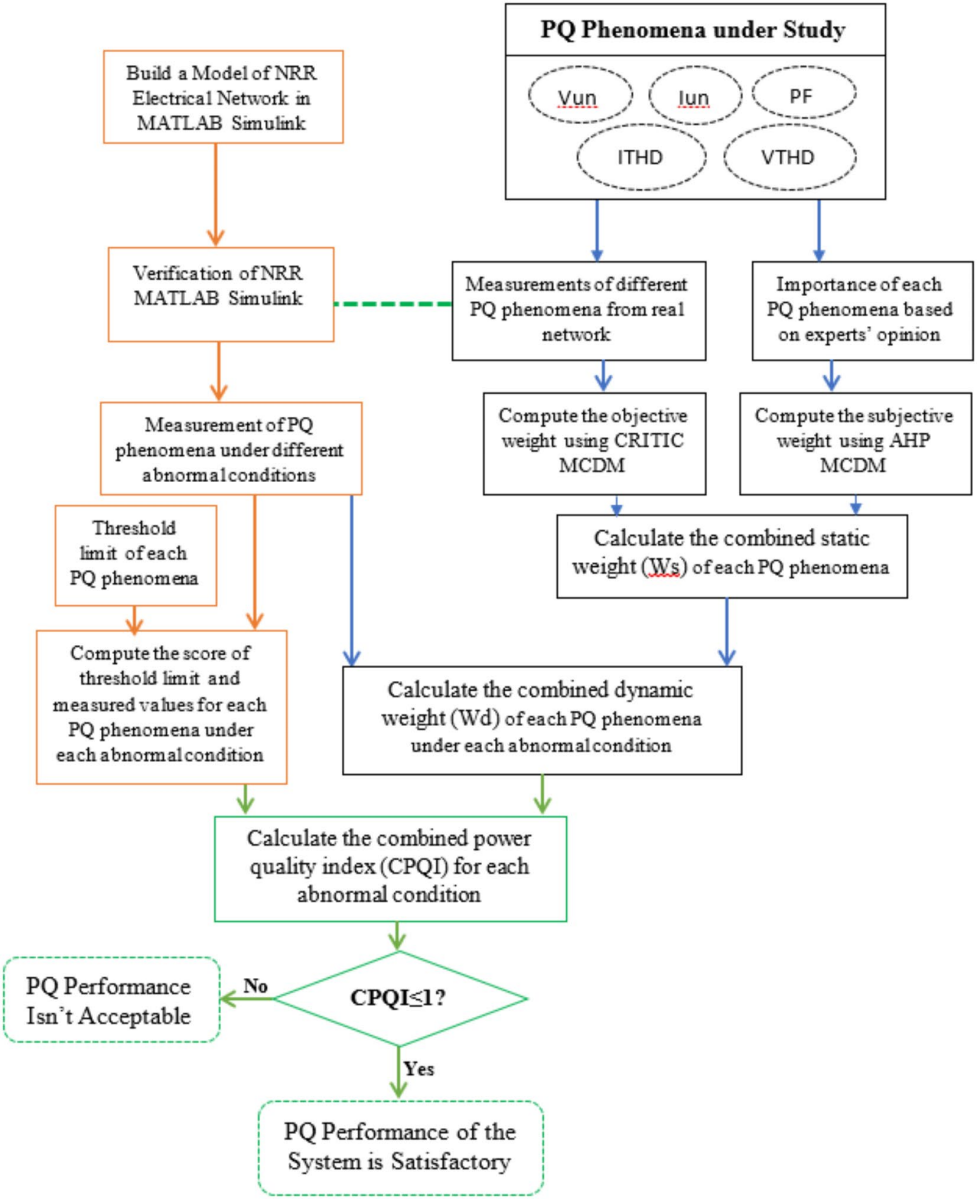
Overall structure of the proposed approach.

This study is organized as follows: After the introduction, an explanation of the study methodology used for the development of a compound PQ index is provided in “Development of the proposed compound power quality index” section. “The indices associated with the PQ phenomenon” section explains and presents the different studied PQ indices. Computing the objective and subjective weights for various PQ phenomena in an NRR electrical system is presented in the “Measurement and analysis of the suggested case study” section. “Description and verification of the NRR Simulink model” Section presents a description of the NRR Simulink model and a simulation of the model under different abnormal conditions. A discussion of the results obtained from the MATLAB Simulink model and computing the combined PQ index for each case is presented in the “Evaluation of the PQ phenomena for the nuclear installation” section. Finally, the “Conclusion” section summarizes the paper and presents the future work, followed by data availability, and then a list of references is subsequently provided.The overall structure of the proposed approach is diagrammatically depicted in Fig. 1.

## Development of the proposed compound power quality index

The single compound PQ index is a term that can be used to express the overall performance of the system in case of integrating more than PQ phenomena in a single index. Also, it can be called as combined power quality index, global power quality index, composite power quality index, unified power quality index, overall power quality index, or others^33,35^, and^37^. This compound PQ index is sufficient and useful for achieving many benefits, such as fast PQ monitoring and assessment, a decrease in the amount of time required for managing big data on PQ events, and well-organized planning in modern restructured power markets^31^.

Decision-making usually involves multiple criteria and alternatives, and the criteria usually have different importance. So that, we need a scientific way to deal with these multiple criteria and alternatives, which is called MCDM methods. As the current study aims to determine a combined power quality index from multiple and contradictory criteria, MCDM is required to ensure a reliable and effective decision. In this section, the process of constructing a combined dynamic weight via AHP and CRITIC is illustrated in detail.

### Subjective weight calculations

The AHP is a method for calculating subjective weights. It is an MCDM technique that can deal with decision-making problems. The AHP implementation steps are as follows^42^:

#### Step 1

**Fig. 2:**
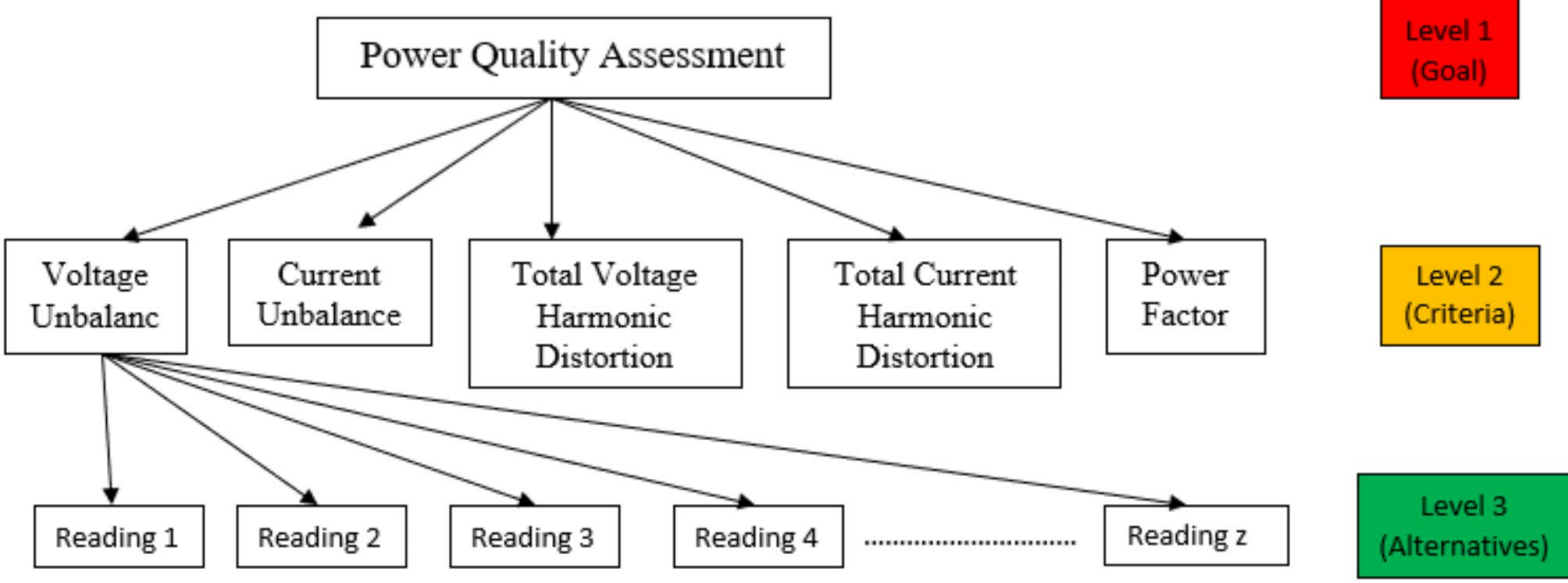
The structure of the AHP approach.

Build a hierarchical structure with a goal at the top level, criteria or attributes at the second level, and alternatives at the third level. Each alternative has its own set of associated criteria (see Fig. 2).

#### Step 2

Create a pairwise comparison matrix to show the relative importance of various criteria concerning the goal. The comparison matrix can be established according to the 1–9 scale method (see Table 2). This matrix length is equal to the number of criteria.

**Table 2:** Relative importance scale of alternatives and criteria^31,42^.

Importance scale	Interpretation	Clarification
1	Equally important	There are two alternatives that favor the goal equally.
2	Slightly more	
3	Moderately important	An activity favored over another
4	Moderate +	
5	Strongly important	Judgment and experience strongly favor one criterion over another
6	Strong +	
7	Very strongly important	Judgment and experience very strongly favor one activity over another
8	Very, very strongly important	
9	Extremely important	An activity is extremely important over another and this is the highest possible order.

#### Step 3

The normalized pairwise comparison matrix is obtained by dividing each element of the column by the sum for that column.

#### Step 4

The weight of each criterion () is calculated by averaging all the elements in the row.

#### Step 5

A consistency test can be performed through the following steps.

- Each value in the comparison matrix column is multiplied by the criteria weight.
- The weighted sum value is calculated by taking the sum of each value in the row.
- The weight ratio of the weighted sum value to the criterion weight for each row is called lambda $\:\lambda\:$; then, $\:{\lambda\:}_{max}$ can be calculated by taking the average of all these values.
- Calculate the consistency index $\:CI$.

1 $$\:CI=\frac{{\lambda\:}_{max}-n}{n-1}$$

where $\:n$ is the number of criteria.

e) The consistency ratio ($\:CR)$ is calculated by dividing $\:CI$ by the random consistency index $\:RI$, and the values of $\:RI$ depend on the number of criteria ($\:n$) of the comparison matrix, as shown in Table 3.

2 $$\:CR=\raisebox{1ex}{$CI$}\!\left/\:\!\raisebox{-1ex}{$RI$}\right.$$

**Table 3:** Random consistency index values for different numbers of criteria^41^.

$n$	1	2	3	4	5	6	7	8	9
$\:RI$	0	0	0.58	0.9	1.12	1.24	1.32	1.41	1.45

If CR < 0.1, then the pairwise matrix is consistent; otherwise, some values in the pairwise matrix need adjusting to make it consistent.

#### Step 6

If the consistency check is passed, calculate the eigenvector corresponding to, which represents the scores obtained by each of the alternatives.

### Objective weight calculation

The objective weights of the criteria are computed in this study based on the CRITIC method. This methodology involves both standard deviation and correlation. The steps of the CRITIC method are as follows^24^:

**Step 1**: Build the main decision matrix: This decision matrix $\:X={\left[{x}_{ij}\right]}_{m\text{*}n}$ contains $\:m$ alternatives and $\:n$ criteria.

**Step 2**: Normalize the decision matrix: This process is based on each criterion’s best and worst value, taking into consideration that for beneficial criteria, the best value is the max value and the worst value is the min value, and vice versa for non-beneficial criteria.

3 $$\:\stackrel{-}{{x}_{ij}}=\frac{{x}_{ij}-{x}_{j}^{worst}}{{x}_{j}^{best}-{x}_{j}^{worst}}$$

where $\:\stackrel{-}{{x}_{ij}}$ is the normalized decision matrix, $\:{x}_{ij}$ is the actual value of alternative $\:i$ for criterion $\:j$, $\:{x}_{j}^{worst}$ is the criterion worst value, and $\:{x}_{j}^{best}$ is the criterion best value.

#### Step 3

Calculate the standard deviation for each criterion using the normalized values.

#### Step 4

Using the linear correlation coefficient between each two criteria, the symmetric matrix of with element can be determined.

#### Step 5

Calculate a measure of the conflict created by criterion for the decision situation defined by the remaining criteria.

4 $$\:\sum\:_{j,\:\:k=1}^{n}\left({1-r}_{jk}\right)$$

#### Step 6

Determine the quantity of the information about each criterion using this formula.

5 $$\:{C}_{j}={\sigma\:}_{j}\text{*}\sum\:_{j,\:\:k=1}^{n}\left({1-r}_{jk}\right)$$

#### Step 7

Determine the objective weight () for each criterion using the following formula.

6 $$\:{W}_{cj}=\frac{{C}_{j}}{\sum\:_{j=1}^{n}{C}_{j}}$$

### AHP-CRITIC combined static weight

Although CRITIC weights provide useful and effective information, they heavily depend on objective data and do not take into consideration expert experience and knowledge wealth when making a decision, which may not be compatible with the reality and understanding of the problem. Therefore, it is necessary to combine the objectivity of the CRITIC weighting and the subjectivity of the AHP method to guarantee the effectiveness and reliability of the obtained weights. The integration of these two methodologies aims to obtain weight that combines experts’ experience and knowledge and the objective variability of the evaluation data. After the calculation of the AHP subjective weight vector $\:{W}_{a}$ and the CRITIC objective weight $\:{W}_{c}$, we can obtain an overall combined static weight $\:{W}_{s}$for n criteria (PQ phenomena) using the following equation^43^:

7 $$\:{W}_{sj}=\frac{{W}_{aj}{W}_{cj}}{\sum\:_{j=1}^{n}{W}_{aj}{W}_{cj}}$$

where $$\:j$$ = 1, 2, 3,…….,$$\:n$$

### Dynamic weight mechanism

In conventional PQ assessment methods, the weight of each PQ phenomenon always remains constant. Suppose that an indicator violates the normal range or standard limit. In this case, it is possible that the overall PQ index still indicates that the electrical system is healthy because this PQ phenomenon weight is relatively small. This situation shows that static weighting methods have inherent disadvantages. Therefore, the dynamic weight approach is proposed in this paper to ensure that the weight of each PQ phenomenon follows the change in operation conditions to assess the actual status of the electrical system. As the static weight is computed depending on the operating conditions of the electrical system, the dynamic weight ($\:{W}_{d}$) must depend on the change in the operating conditions of the NRR relative to the standard limit. Thus, the dynamic weight can be calculated from the static weight considering the change in the status of the electrical system relative to the standard limit for each PQ phenomenon using the following equation:

8 $$\:{W}_{dj}={W}_{sj}+{W}_{sj}\times\:\frac{{{F}_{m}}_{j}-{{F}_{t}}_{j}}{{{F}_{t}}_{j}}$$

where $\:{{F}_{m}}_{j}$ is the current measurement value of the PQ phenomenon and $\:{{F}_{t}}_{j}$ is the threshold (standard) limit of each PQ phenomenon. Then, the weight $\:{W}_{dj}$ needs to be normalized as follows:

9 $$\:{W}_{dj}=\frac{{W}_{dj}}{\sum\:_{j=1}^{n}{W}_{dj}}$$

## The indices associated with the PQ phenomenon

The degree of effect for one PQ phenomenon is generally different from that for another^41,44^. Therefore, MCDM methods can be used to identify the importance and weight of each PQ phenomenon. overall PQ performance is usually based on a number of PQ indices. Among the most important PQ phenomena are voltage unbalance ($\:{V}_{Un}$), current unbalance ($\:{I}_{Un}$), current harmonic distortion ($\:{I}_{THD}$), voltage harmonic distortion ($\:{V}_{THD}$), and the power factor ($\:PF$). These phenomena are more important than other power quality indicators because of the extent to which they impact the operation of the electrical network, as mentioned in Table 4. Potential problems resulting from these effects and their impact on safety are highly influential when choosing the PQ phenomenon under study.

**Table 4:** The main defects of the five PQ indices.

Phenomena	Demerits
voltage unbalance ($\:{V}_{Un})$	• Lead to vibration in motor developed torque. • Leads to acoustic noises in the mechanical equipment. • Miss operation for the facility. • High temperatures. • Reduction in the life of equipment.
current unbalance ($\:{I}_{Un})$	
Voltage harmonic distortion ($\:{V}_{THD})$	• higher electricity bills • Increased Heating • Reduction in the life of equipment. • Resonance Issues • sensitive equipment malfunction • Electromagnetic Interference (EMI) with Communication Systems • Overloading of Neutral Conductors
Current harmonic distortion ($\:{I}_{THD})$	
Low power factor ($\:PF$)	• Decrease efficient power. • higher electricity bills

### Current and voltage harmonics

The nonlinearity of loads in the electrical network leads to harmonics distortion. One of the major problems associated with harmonic distortion is the harmonic resonance, as it can lead to equipment damage or malfunction. Additionally, harmonics can cause equipment overloading, an increase in losses, and equipment malfunction. The most commonly used harmonic indices are $\:{I}_{THD}$ and $\:{V}_{THD}$^24^. $\:{V}_{THD}$ can be obtained from the harmonic voltage $\:{v}_{h}$using the following equation^33^:

10 $$\:\:{V}_{THD}=\frac{\sqrt{\sum\:_{h=2}^{{\infty\:}}{V}_{h}^{2}}}{{V}_{1}}\text{*}100\text{\%}$$

Additionally, $\:{I}_{THD}$ can be obtained from the harmonic current $\:{I}_{h}$, which results from the operation of nonlinear devices on the electrical network and can be calculated using the following equation^33^:

11 $$\:{I}_{THD}=\frac{\sqrt{\sum\:_{h=2}^{{\infty\:}}{I}_{h}^{2}}}{{I}_{1}}\text{*}100\text{\%}$$

According to the IEEE standard 519, the threshold limits of both $\:{V}_{THD}$ and $\:{I}_{THD}$ are set at 5%.

### Power factor

It is a measure of effectiveness for electrical power and can be calculated by dividing the active power by the apparent power. A high PF benefits both the utility and the customer, while a low PF indicates poor use of electricity. Under ideal conditions, the current and voltage are “in phase,” and the PF is unity (“100%”). In the case of existing inductive loads (motors) in a power circuit, a PF less than 100% can occur^24^. For good performance of the network, the PF value should be greater than 0.95 and less than 100%^33^.

### Voltage and current imbalance

The electrical network performance in terms of voltage imbalance ($\:{V}_{un})$can be calculated based on the following equation^32^:

12 $$\:\%\:{V}_{un}=\:\frac{82*\sqrt{{{V}_{abe}}^{2}+{{V}_{bce}}^{2}+{{V}_{cae}}^{2}}}{average\:line\:voltage}$$

where $\:{V}_{abe}$ is the difference between the line voltage $\:{V}_{ab}$ and the average line voltage. A $\:{V}_{un}$of 2% is adopted as the threshold, as suggested by standard EN 50,160^45^. One voltage imbalance source is the unequal apparent power in each phase of the electrical network. The proposed nuclear facility electrical system model is implemented in MATLAB Simulink to simulate voltage imbalance by switching unbalanced loads on three phases at a certain bus^32^. In addition, the current imbalance is calculated using Eq. 13.

13 $$\:\%\:{I}_{un}=\frac{{I}_{max}-{I}_{av}}{{I}_{av}}*100$$

14 $$\:{I}_{av}=\frac{{I}_{a}+{I}_{b}+{I}_{c}}{3}$$

where $\:{I}_{un}\%$ is the current imbalance percentage and $\:{I}_{max}$ is the phase current with maximum deviation from the average current ($\:{I}_{av}$).

### Compound power quality index formulation

Following the computation of the dynamic weight and score of each PQ phenomenon, Eq. 15 formulates the CPQI of an electrical system^33^.

15 $$\:CPQI=\frac{\sum\:_{j=1}^{n}{W}_{dj}{{S}_{m}}_{j}}{\sum\:_{j=1}^{n}{W}_{dj}{{S}_{t}}_{j}}$$

16 $$\:{{S}_{m}}_{j}=\frac{{F}_{j}}{{{F}_{m}}_{j}+{{F}_{t}}_{j}}$$

17 $$\:{{S}_{t}}_{j}=\frac{{{F}_{t}}_{j}}{{{F}_{m}}_{j}+{{F}_{t}}_{j}}$$

where $\:{{S}_{m}}_{j}$denotes the score for the measured $\:{j}_{th}$ PQ phenomenon, $\:{{S}_{t}}_{j}$denotes the score for the threshold $\:{j}_{th}$ PQ phenomenon, $\:t$ indicates the value of the threshold limit, and $\:m$ indicates the value of the measurement.

A CPQI value less than unity indicates that the PQ performance is healthy. A unity value of the CPQI indicates that the PQ performance is on the verge of the threshold, and a value greater than unity signifies unhealthy performance for the system power quality^33^.

## Measurement and analysis of the suggested case study

### Overview of the NRR electrical loads

**Fig. 3:**
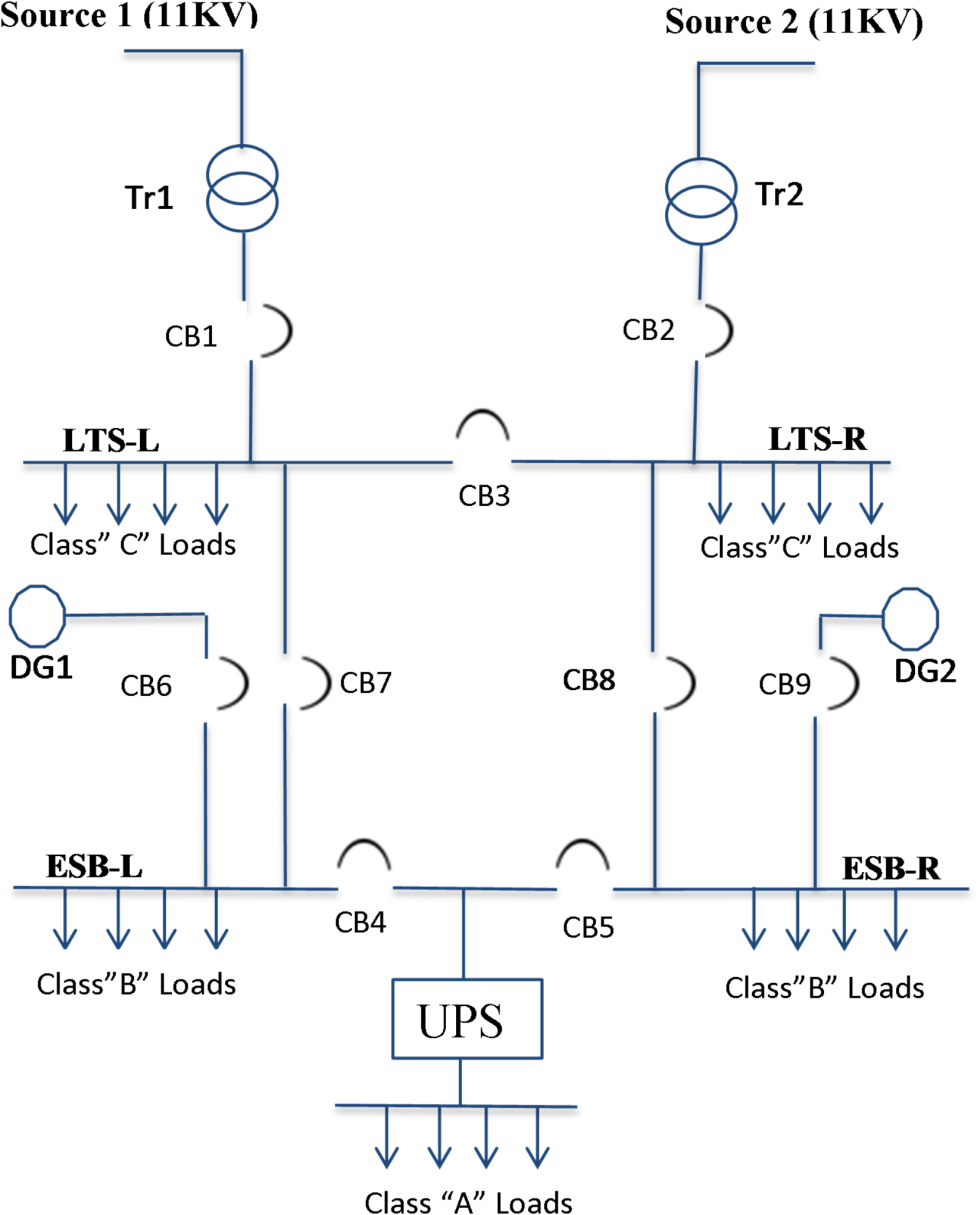
NRR electrical system single-line diagram.

To ensure safe and reliable operation for nuclear installation, sufficient power must be provided with the required quality to the systems and equipment in this nuclear facility. Therefore, the electrical power system is considered one of the most important systems in the nuclear facilities. The NRR electrical loads, as shown in Fig. 3, are classified according to the following categories in Table 5.

**Table 5:** Classification of electrical loads for the NRR.

Class of load	Description	Requirements	Notes
A	Loads those are essential from view point of safety.	An uninterruptible AC power supply (UPS).	The capacity of NRR UPS is 15 KVA with an autonomy of 30 min. It meets all required demand load of class “A”.
B	Loads whose reconnection to the system is convenient after the interruption of electrical power supply from the external lines.	Two sources for feeding: the power plant and the normal power supply.	The power plant is about two DG with 300 KVA each. It provides adequate AC power for supplying class “B” and class “A” when the external lines are unavailable.
C	The loads which allow interruption of the supply for a definite time.	They fed from the normal power supply.	The electrical power system of NRR is fed by two independent sources from two different substations.

### Power quality measurements for NRR and calculated CRITIC weights

The NRR is affected by disturbances in the electrical network. The PQ issue is affected mainly by different abnormal conditions, such as disturbances from consumer devices and networks, disturbances from utility feeding systems, nonlinearities of loads and devices, or the selection of unsuitable sites for distribution and transmission lines. Measurements for different PQ phenomena were taken at the NRR LTS-L busbar during half-load operation for this NRR. Then, based on the equations in the “Objective weight calculation” section and the CRITIC MATLAB code, PQ phenomenon weights were computed (see Table 6)^24^.

**Table 6:** PQ phenomena objective weights of an NRR facility.

PQ phenomena	$\:{I}_{un}$	$\:{V}_{un}$	$\:PF$	$\:{V}_{THD}$	$\:{I}_{THD}$
CRITIC Weights	0.242	0.213	0.18	0.204	0.161

### Computing the weights for the NRR PQ phenomena based on AHP

The AHP can be applied to the NRR network to determine the weight of each PQ phenomenon considering three general classes of influence at the bus under study. These classes include miss of operation, life reduction, and losses^46^. The AHP’s first step is to construct the decision matrix based on experts’ opinions. Three matrices are built, one of which represents the order of PQ phenomena according to its effect on NRR miss of operation, the other concerned with the ordering and its effect on cost, and the third shows the ordering considering its impact on the equipment life reduction in the facility (see Tables 7, 8 and 9). Then, as shown in Table 10, using the AHP methodology, the weight under each condition and the overall weight for each PQ phenomenon can be obtained:

**Table 7:** Decision matrix corresponding to the Miss of operation.

	$\:{V}_{Un}$	$\:{I}_{Un}$	$\:{V}_{THD}$	$\:{I}_{THD}$	$\:PF$
$\:{V}_{Un}$	1	2	4	5	7
$\:{I}_{Un}$	0.5	1	3	4	6
$\:{V}_{THD}$	0.25	0.333333	1	2	4
$\:{I}_{THD}$	0.2	0.25	0.5	1	3
$\:PF$	0.142857	0.166667	0.25	0.333333	1

**Table 8:** Decision matrix corresponding to cost.

	$\:PF$	$\:{I}_{THD}$	$\:{V}_{THD}$	$\:{I}_{Un}$	$\:{V}_{Un}$
$\:PF$	1	3	4	6	7
$\:{V}_{THD}$	0.333333	1	2	4	5
$\:{I}_{THD}$	0.25	0.5	1	3	4
$\:{I}_{Un}$	0.166667	0.25	0.333333	1	2
$\:{V}_{Un}$	0.142857	0.2	0.25	0.5	1

**Table 9:** Decision matrix corresponding to life reduction.

	$\:{I}_{Un}$	$\:{V}_{Un}$	$\:{I}_{THD}$	$\:{V}_{THD}$	$\:PF$
$\:{I}_{Un}$	1	2	4	5	7
$\:{V}_{Un}$	0.5	1	3	4	6
$\:{I}_{THD}$	0.25	0.333	1	2	4
$\:{V}_{THD}$	0.2	0.25	0.5	1	3
$\:PF$	0.143	0.167	0.25	0.333	1

**Table 10:** Overall weights of PQ phenomena computed by the AHP.

PQ Phenomena	Class Name			
	Mis of operation	cost	life reduction	Overall
$\:{I}_{Un}$	0.294	0.069	0.445	0.269
$\:{V}_{Un}$	0.445	0.046	0.294	0.262
$\:{I}_{THD}$	0.086	0.236	0.133	0.152
$\:{V}_{THD}$	0.133	0.153	0.086	0.124
$\:PF$	0.042	0.496	0.042	0.193
Largest eigenvalue ($\:{\lambda\:}_{max}$)	5.139464	5.139719	5.139	-
Consistency ratio ($\:CR$)	0.031004	0.031061	0.031	-
Consistency check	Passed	Passed	Passed	Passed

### Calculating the AHP-CRITIC combined static weight for the NRR

**Fig. 4:**
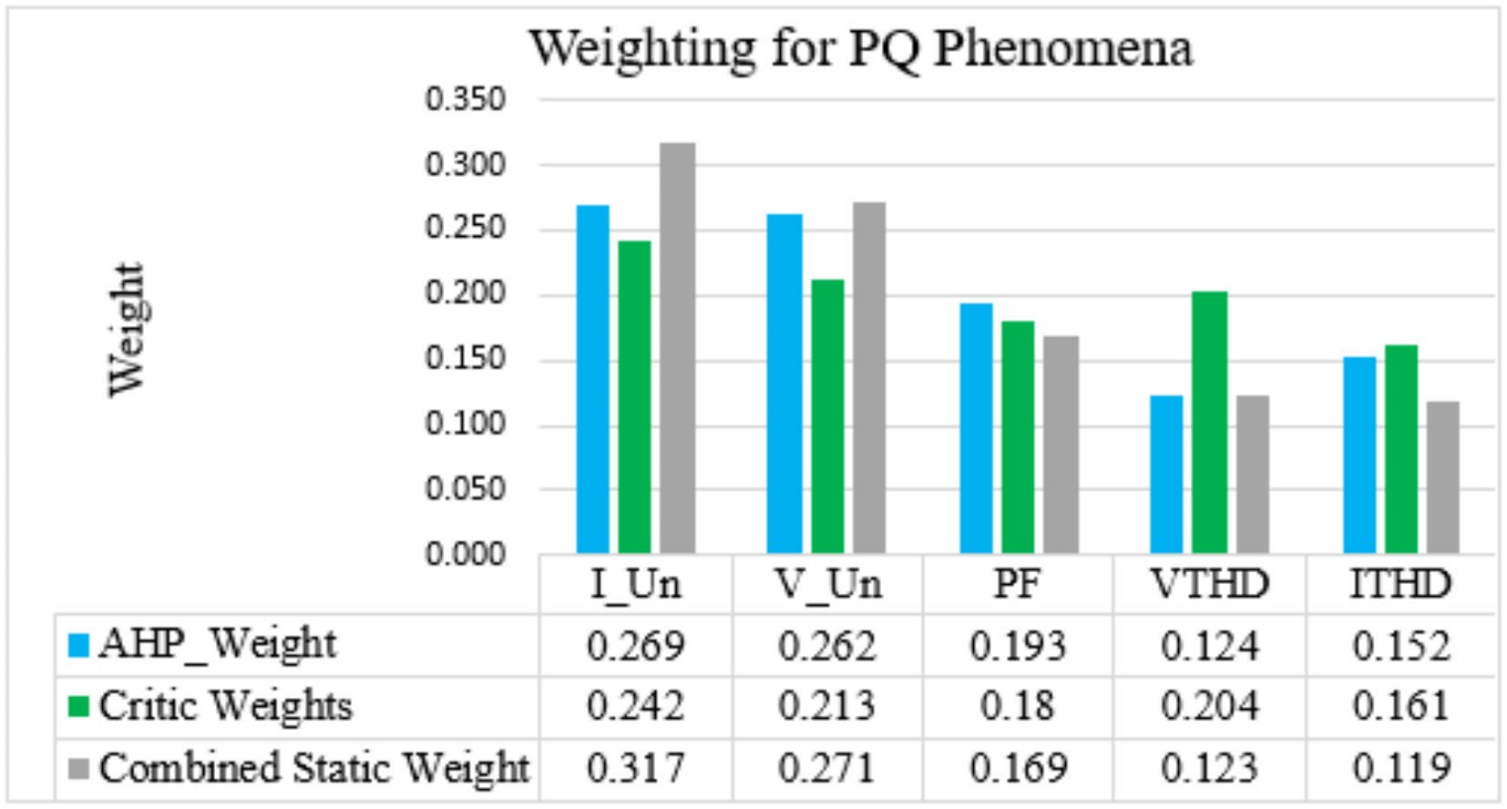
Weight comparison chart.

The combined static weight $\:{W}_{s}$ can be calculated based on the equation in the “AHP-CRITIC combined static weight” section (see Table 11; Fig. 4).

**Table 11:** PQ phenomena weights computed by AHP, CRITIC and AHP-CRITIC.

	$\:{I}_{Un}$	$\:{V}_{Un}$	$\:PF$	$\:{V}_{THD}$	$\:{I}_{THD}$
Weights AHP ($\:{W}_{a})$	0.269	0.262	0.193	0.124	0.152
Weights Critic ($\:{W}_{c})$	0.242	0.213	0.18	0.204	0.161
Combined Static Weight ($\:{W}_{S})$	0.317	0.271	0.169	0.123	0.119

As shown in Fig. 4, based on both the AHP and CRITIC weighting methods, all the studied PQ phenomena are important for this nuclear installation, with greater importance given for the imbalance. The main and important equipment in the NRR under study is the reactor coolant pumps, which transfer and remove the generated heat in the reactor core by providing a forced coolant flow to prevent fire or meltdown in this reactor core^47^. This NRR operates at its full power (22 MW thermal) with two pumps (2000 m^3^/h three-phase motors) for cooling^48^, and there are many single-phase loads in this nuclear installation. In addition, operating three-phase motors under unbalanced conditions causes problems such as vibrations, overheating, insulation damage, noise, an increase in voltage drop, and in-line power losses due to an increase in line currents^49^. To ensure the safety of NRRs, an unbalanced PQ phenomenon must have great importance^24^. This means that the obtained weights are compatible with the safety of this nuclear facility.

## Description and verification of the NRR Simulink model

The NRR electrical system is supplied from two different substations by two independent sources at medium voltage levels to maintain good reliability. Each source feeds a separate transformer through a circuit breaker. Both the transformer and its busbar section in the plant can carry the total demand power^50^. One of the typical IEEE standard configurations, which is called the secondary selective system, is used in the radial feeding of electricity for the NRR under study^51^. The proposed system is described in Table 12.

**Table 12:** The rating of the proposed electrical system.

Device	Rating
Tr1	Dy 11, 50 Hz, 11 kV/0.4 kV, 2000 kVA
Tr2	Dy 11, 50 Hz, 11 kV/0.4 kV, 2000 kVA
Class C_ Left Busbar	1684.56 A, 671.26 KW
Class C_ Right Busbar	1602.34 A, 838.29 KW
Class B_ Left Busbar	263.42 A, 107.45 KW
Class B_ Right Busbar	88.59 A, 36.07 KW
Class A	73.8 A, 33.25 KW
UPS	45 A, 19.25 KW, 15 KVA, Autonomy 30 min
DG1	300 KVA
DG2	300 KVA

**Fig. 5:**
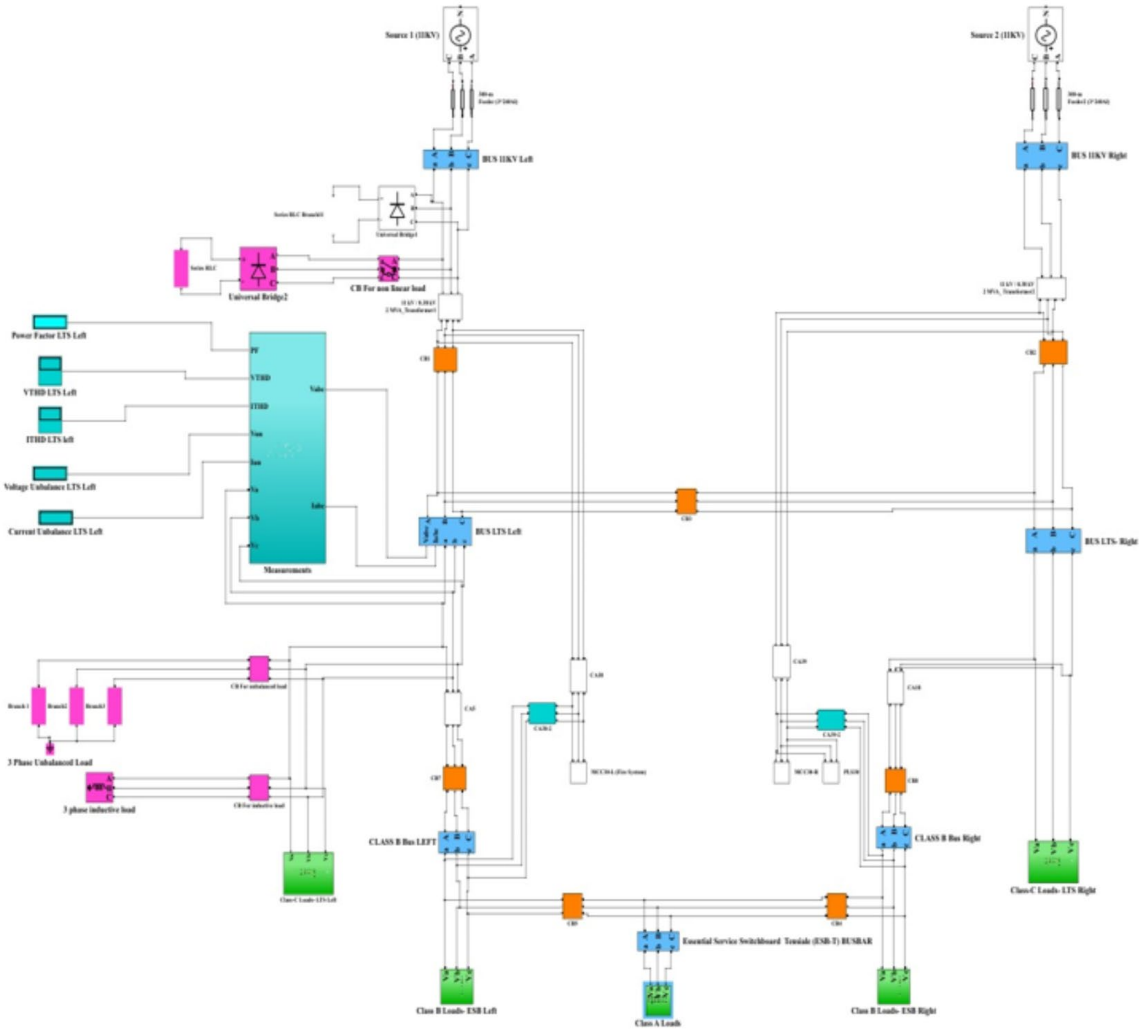
MATLAB Simulink model for the NRR electrical system.

**Fig. 6:**
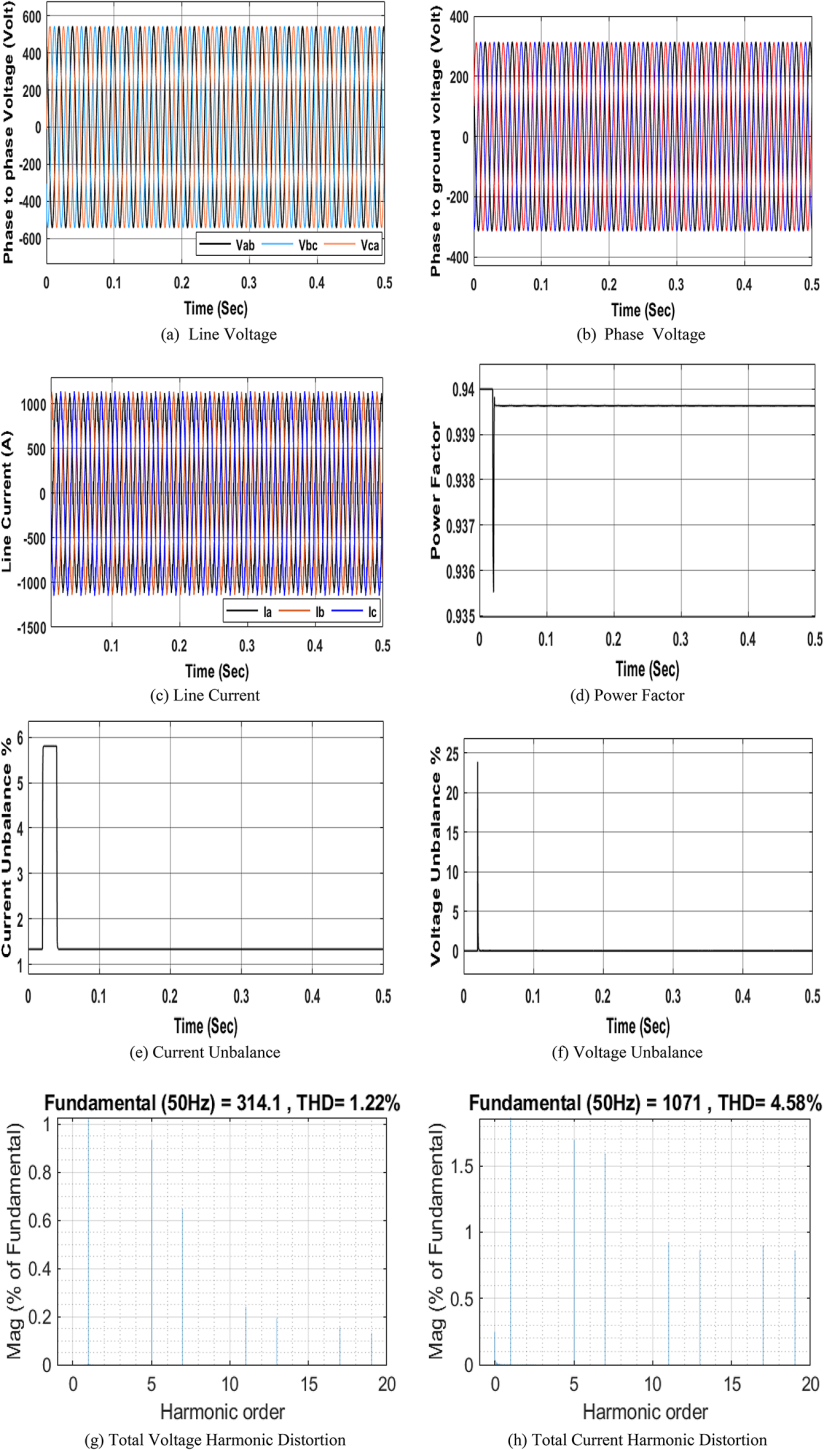
Simulation of PQ phenomena under half-load normal operating condition of the NRR.

The NRR model was constructed in MATLAB/Simulink with all its components (Fig. 5). Then, to ensure credibility and validity, this NRR model is subjected to conditions similar to those under which the measurements were taken from the real electrical network. These conditions included reducing or disconnecting some loads, creating some imbalance in the loads, and adding a harmonic source to the system to ensure a response for both voltage, current, voltage unbalance, power factor, and total harmonic distortion similar to that obtained from the real NRR electrical network. Figure 6 shows a comparison of different electrical parameters between measurements from the real NRR network and from the constructed MATLAB Simulink model.

By comparing the results obtained from the MATLAB model with the real measurements, it is found that the results from the model fall within the range of the results obtained from the real measurements. This shows that the performance of this model is very close to that of a real NRR facility (see Table 13.).

**Table 13.** Comparison between measurements from the real NRR network and MATLAB Simulink model.

**Variables**	**Measurement**		**NRR MATLAB Model**	**Variables**	**Measurement**		**NRR MATLAB Model**
	**Min**	**Max**			**Min**	**Max**	
V_a_	220	225	222.1	$\:{V}_{{THD}_{a}}$	0.59	1.59	1.224
V_b_	220	226.5	221.9	$\:{V}_{{THD}_{b}}$	0.62	1.59	1.228
V_c_	220	226.5	221	$\:{V}_{{THD}_{c}}$	0.62	1.61	1.232
I_a_	468	766	757.8	%$\:{V}_{THD}$	-	-	1.22
I_b_	471	772	770.7	$\:{I}_{{THD}_{a}}$	1.91	4.74	4.576
I_c_	459	780	775.7	$\:{I}_{{THD}_{b}}$	1.69	4.58	4.499
PF	0.92	0.96	0.94	$\:{I}_{{THD}_{c}}$	1.63	4.74	4.467
V_Un_	0.01	0.09	0.02266	%$\:{I}_{THD}$	-	-	4.58
I_Un_	0.32	1.81	1.34				

PQ phenomena were monitored at the NRR low-tension switchboard (LTS) at the left busbar under different abnormal operating conditions, including a three-phase nonlinear load to simulate harmonics, switching an unbalanced load to simulate unbalance, and adding an inductive load to simulate the change in the power factor.

### Simulation Model for three-phase nonlinear load

**Fig. 7:**
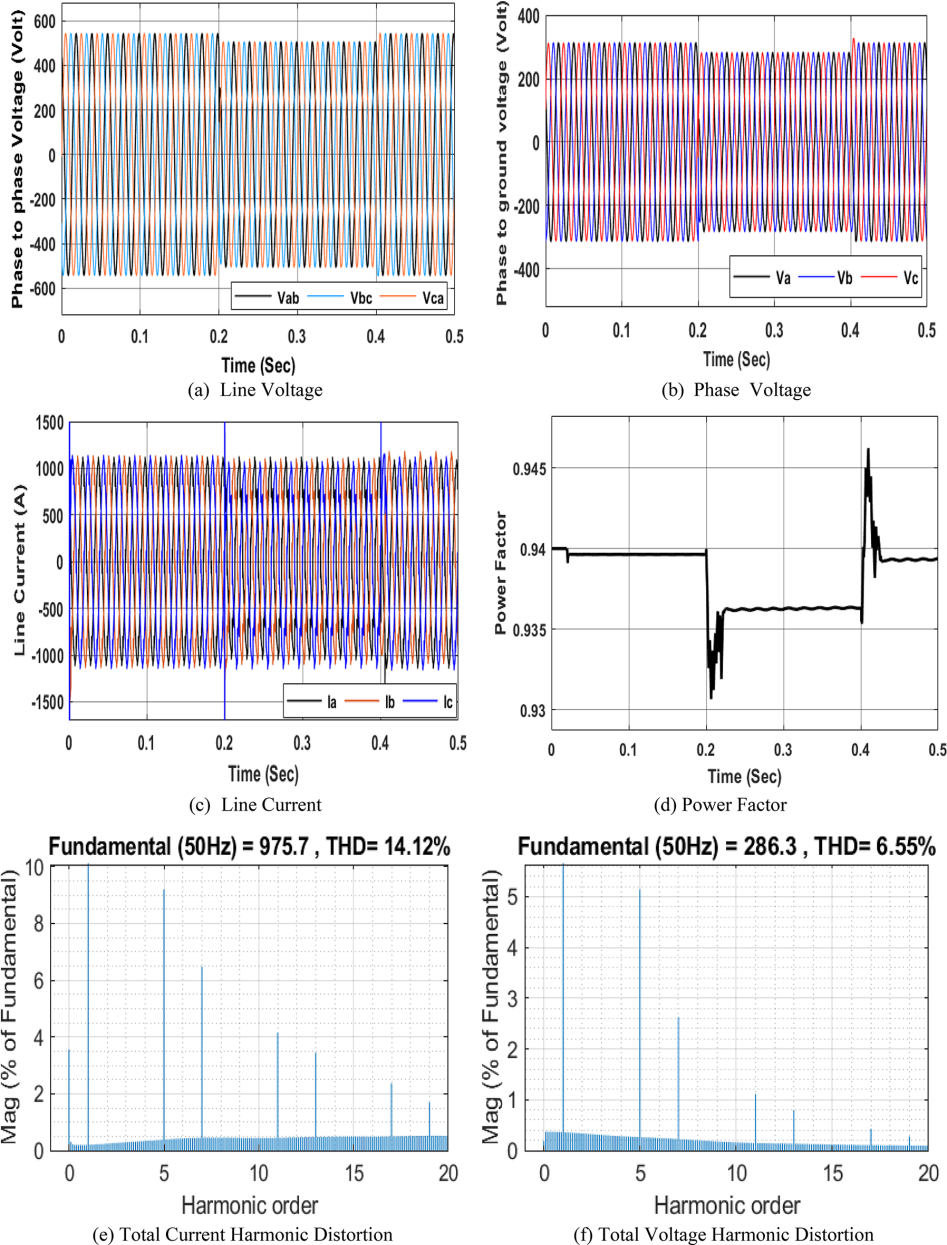
Simulation of PQ phenomena due to nonlinear load.

The nonlinear load model is used to simulate both voltage and current harmonic distortion. The nonlinear model involves a three-phase universal bridge diode connected across the series inductive-resistive branch. The three-phase universal bridge has a snubber resistance *R* = 9 Ω, infinite snubber capacitance, internal resistance (Ron) = 2e-2 Ω, internal inductance (Lon) = 0, and forward voltage = 0 V. The series inductive resistive load across the universal bridge consists of 1 mH inductance and 5 Ω resistance. This nonlinear load is connected after the NRR 11 KV/400 V left transformer, and readings are taken at the LTS left busbar for a duration of 0.2 to 0.4 s. Figure 7 shows the different performances corresponding to the NRR network simulation by adding the three-phase nonlinear model. It is evident that from 0.2 to 0.4 s, the power factor decreases to 0.9363 as shown in Fig. 7d, and both $\:{I}_{THD}$ and $\:{V}_{THD}\:$ increase to 14.12% and 6.55%, respectively as shown in Fig. 7e and f, due to the existence of a nonlinear load, which injects harmonics into the system.

### Simulation Model for an unbalanced load

**Fig. 8:**
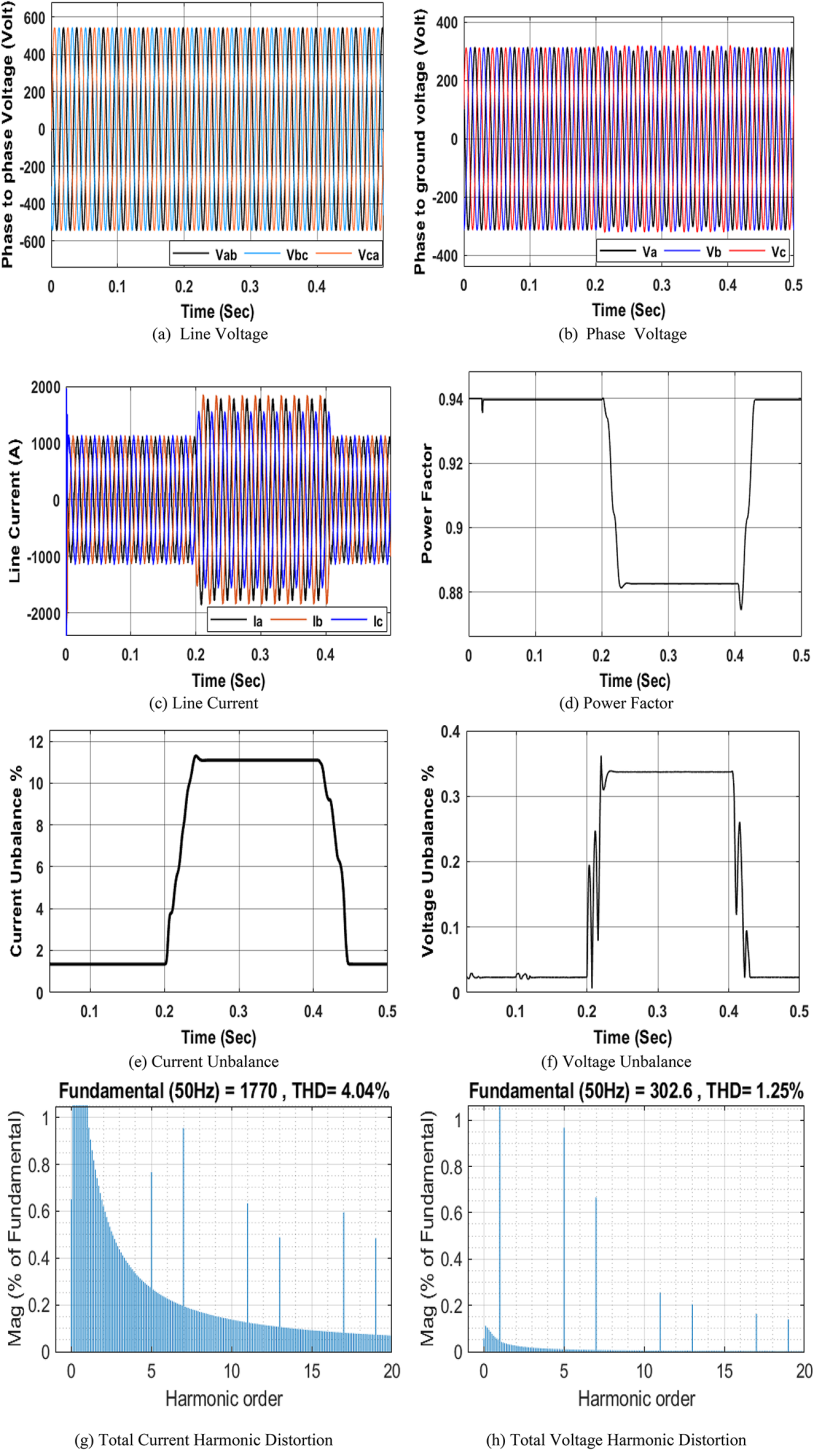
Simulation of the PQ phenomena due to an unbalanced load.

The unbalanced load switching on the left busbar of the NRR model has been used to simulate.

and $\:{I}_{Un}$ caused by the unbalanced load on the electrical system. The unbalanced load switching model has three different inductive resistive loads in each phase; their ratings are 0.25 ohm, 0.9 mH; 0.3 ohm, 0.92 mH; and 0.75 ohm, 1 mH. The three different series R-L branches are connected at the three phases on the LTS left busbar for a duration of 0.2 to 0.4 s to simulate voltage and current imbalance. Figure 8 shows the different waveforms corresponding to the NRR network simulation using the unbalanced load model. The main causes of imbalance are unsymmetrical loads and unsymmetrical distribution systems. The obtained results show that an unbalanced load leads to an increase in the voltage and current. Additionally, it affects the power factor. It is obvious that under stable system conditions (before 0.2 s and after 0.4 s), the current imbalance and voltage imbalance are close to 1.34 and 0.02266, respectively. From 0.2 to 0.4 s, due to the unbalanced load, the current and voltage imbalances increase to 11.09 and 0.3372, respectively as shown in Fig. 8e, 8f.

### Simulation Model for adding an inductive load

**Fig. 9:**
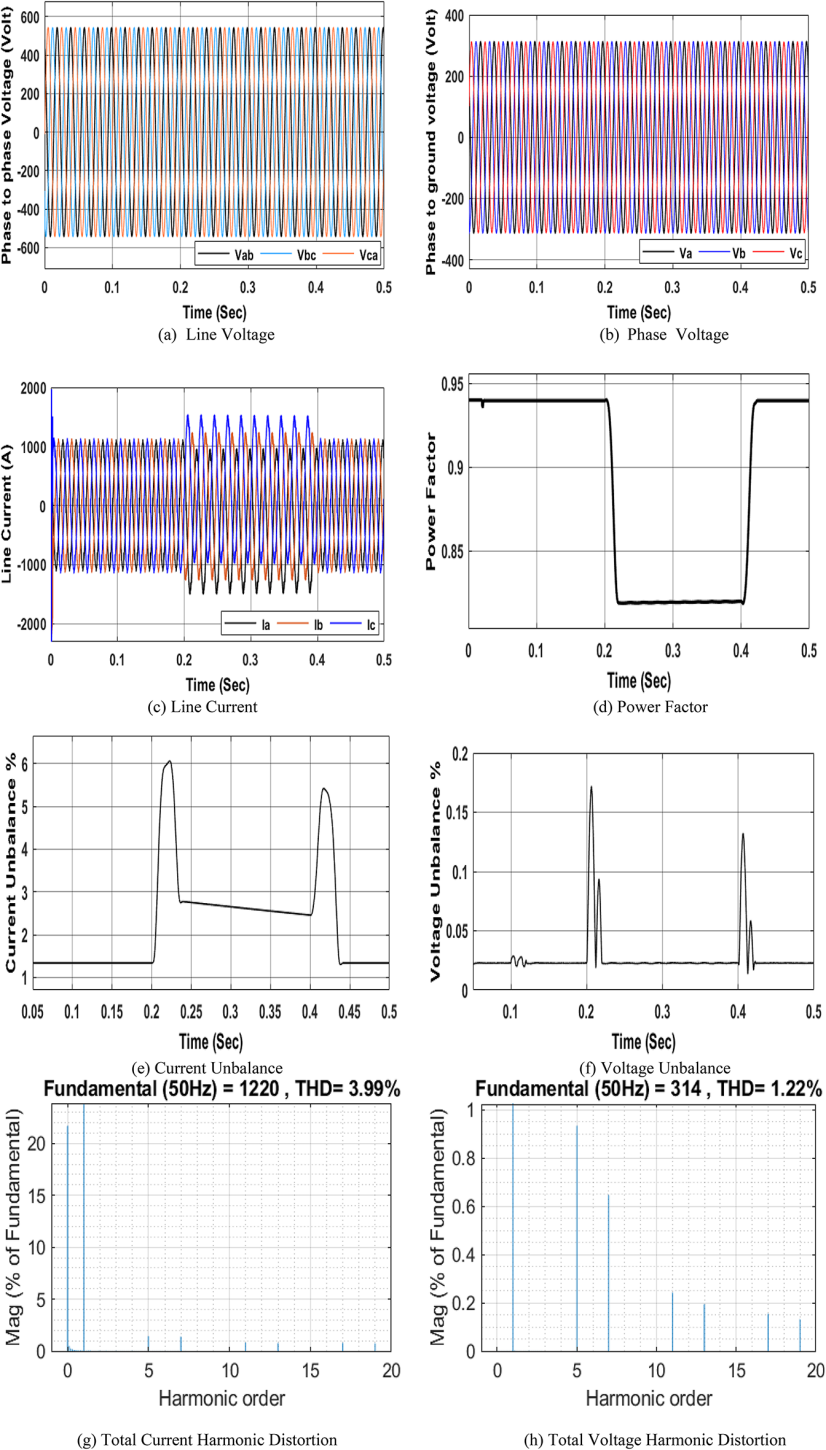
Simulation of the PQ phenomena due to the inductive load.

An inductive load is added at the NRR LTS left busbar for a duration of 0.2 to 0.4 s to simulate the change in the power factor. The rating of this inductive load is 150 kVAR. Different waveforms are monitored at the LTS left busbar. Figure 9 shows the different waveforms corresponding to the NRR network, simulated under an inductive load. Adding this inductive load causes a decrease in the system PF. A low PF can lead to an increase in losses, a decrease in system efficiency, and an increase in costs. Additionally, inductive loads cause fluctuations and drops in voltage, which can affect the operation of other electrical equipment and devices. It is obvious that under stable conditions of the system (before 0.2 s and after 0.4 s), the power factor is close to 0.94. However, when an inductive load is added (from 0.2 to 0.4 s), the power factor decreases to 0.8195 as shown in Fig. 9d.

### Simulation Model for adding nonlinear, unbalanced, and Inductive loads

**Fig. 10:**
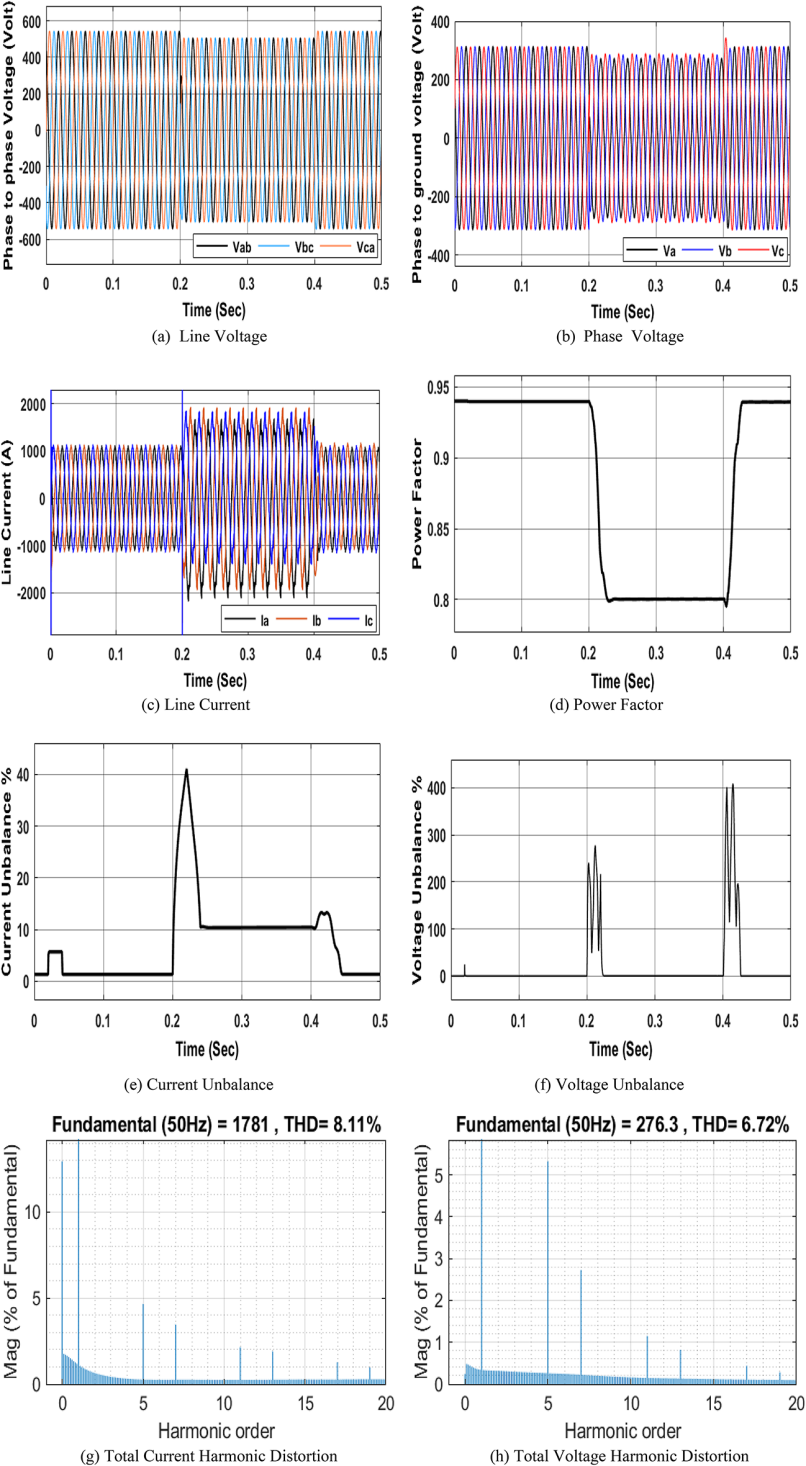
Simulation of PQ phenomena due to nonlinear, unbalanced, and inductive loads.

This subsection presents the simulation results obtained by adding the previous three abnormal conditions (nonlinear, unbalanced, and inductive loads) to the NRR electrical network for a duration of 0.2 to 0.4 s. The exact parameter used for these abnormal conditions is written in the “Description and verification of the NRR Simulink model” Section, and the configuration of these abnormal conditions is presented in Fig. 5. Different analyses were performed at the LTS left bus bar. Figure 10 shows the different behaviors corresponding to the NRR network, simulated after adding the three abnormal conditions together. The obtained results showed that adding nonlinear, unbalanced, and inductive loads together to the NRR electrical system model leads to a decrease in PF and an increase in $\:{V}_{THD}$ and $\:{I}_{THD}$. Additionally, this leads to an increase in and $\:{I}_{Un}$ .

To facilitate the comparison and analysis for the measurements of PQ indices and their distances from the standard limit, the measured values under each abnormal condition were collected and combined with the threshold limit in Table 14.

**Table 14:** The threshold limit and measured values of PQ indices for different abnormal conditions.

	$\:{I}_{Un}$	$\:{V}_{Un}$	$\:PF$	$\:{V}_{THD}$	$\:{I}_{THD}$
Threshold [[31], [32], [33], [52], [53], [54],	5	2	0.95	5	5
Measured for Nonlinear load	1.281	0.02154	0.9363	6.55	14.12
Measured for Unbalanced load	11.09	0.3372	0.8826	1.25	4.04
Measured for Inductive load	2.546	0.023	0.8195	1.22	3.99
Measured for Non-Linear, Un Balanced, and Inductive Loads	10.42	0.3099	0.8003	6.72	8.11

## Evaluation of the PQ phenomena for the nuclear installation

**Fig. 11:**
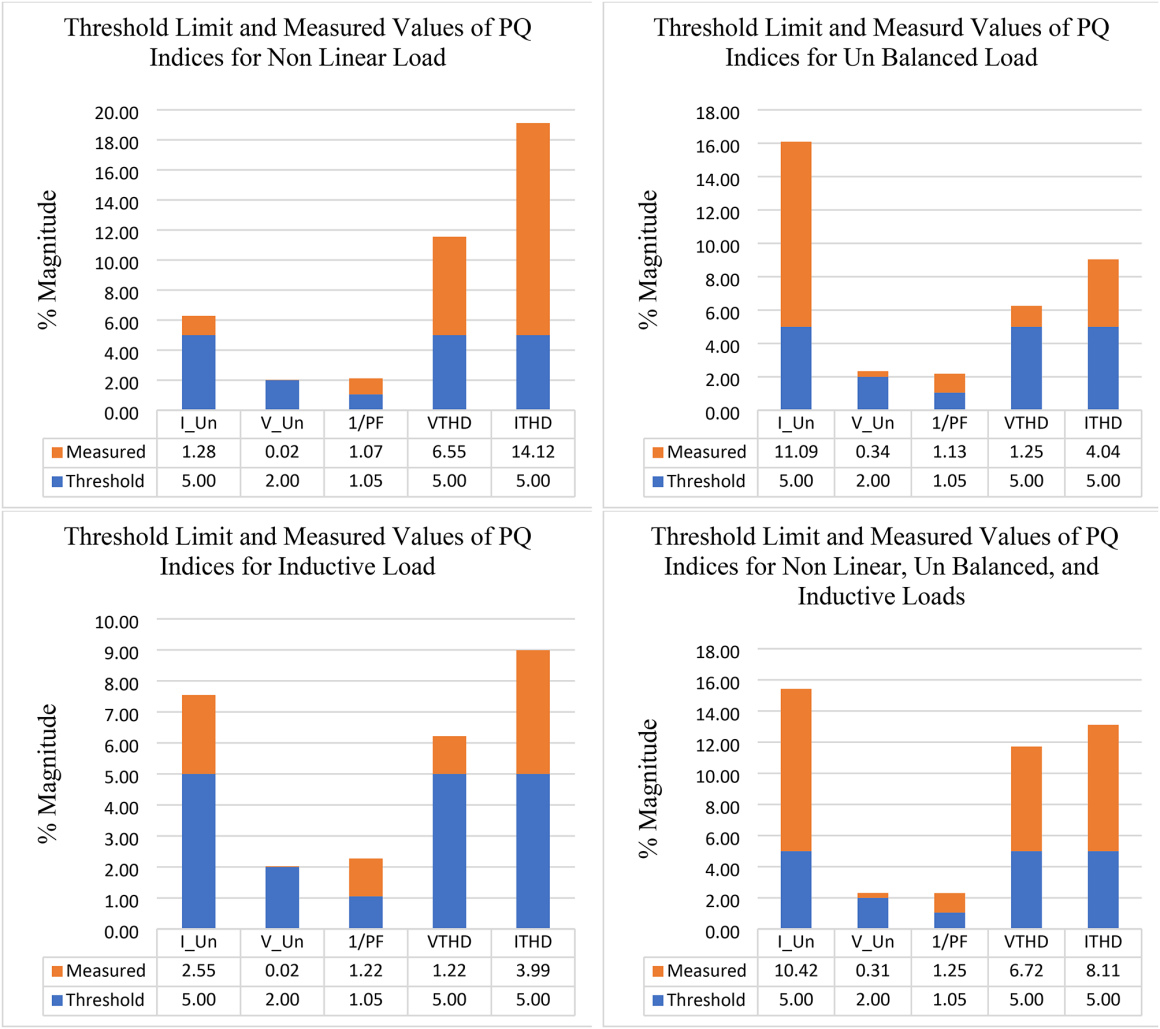
Data of the threshold limit and measured values for PQ indices under different abnormal conditions.

Under the three abnormal conditions, Fig. 11 illustrates the measured values of the PQ indices compared with the threshold limit values, and this figure presents the performance of the NRR under each case and its distance from the standard limit.

**Fig. 12:**
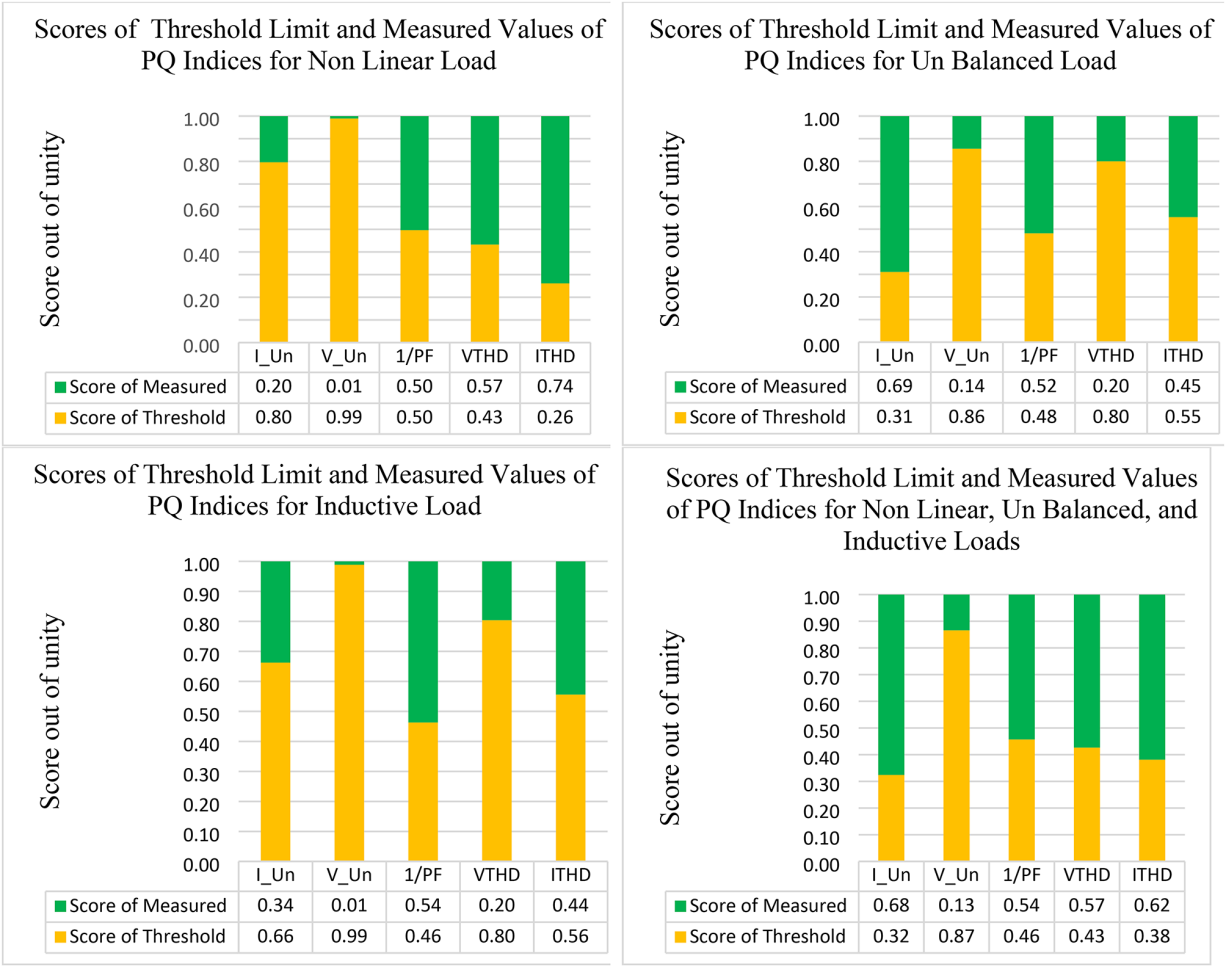
Computed scores for the threshold limit and the measured of PQ indices under different abnormal conditions.

Following the steps of taking measurements, the computed scores for both threshold limits and measured values of all PQ indices are calculated (see Fig. 12).

**Fig. 13:**
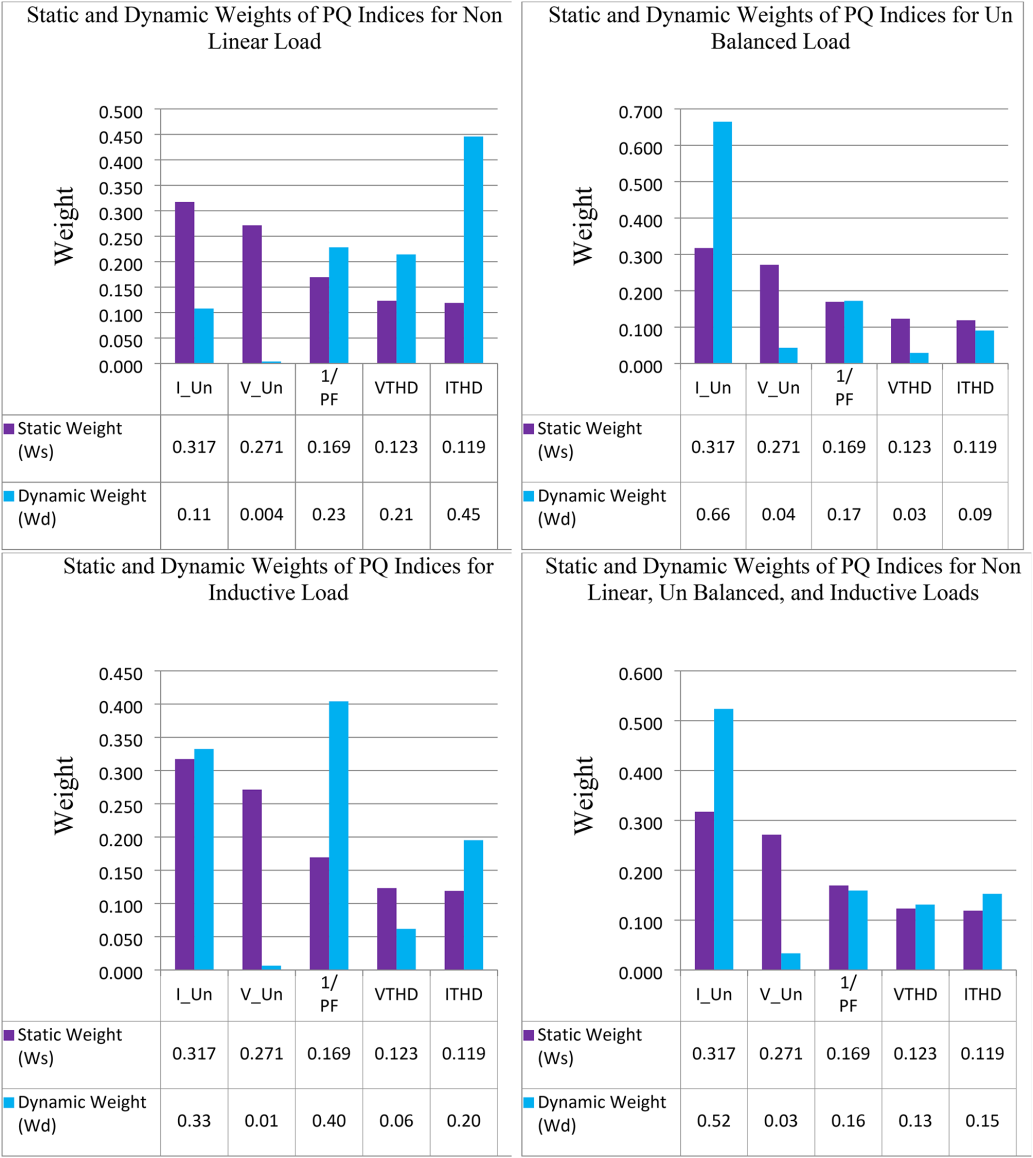
Static and dynamic weights for PQ indices under different abnormal conditions.

The dynamic weights for each PQ phenomenon under each abnormal condition were obtained by using Eqs. 8 and 9 (see Fig. 13). By examining the volatility of the measured values from the threshold limit, we find that the dynamic weight follows the change that occurs in the power quality and gives high importance to the PQ phenomena that access or exceed the standard limit. For example, by studying $\:{I}_{THD}$ under the case of adding a nonlinear load, it was found that the value of this PQ phenomenon is 14.12% compared with the standard limit, which is 5%. Therefore, the score for this measured value is 74%, and the score for the threshold is 26%. Consequently, when the dynamic approach was applied to this case, the weight for this PQ phenomenon increased from 0.119 to 0.45. This means that, using the dynamic weight approach, the importance of PQ phenomena not only depends on a certain condition but also changes with the change in the operating condition of the electrical network.

After computing the scores for both the measured and standard limits, the overall compound PQ index can be obtained from Eq. 15 based on combined dynamic AHP-CRITIC weights and the score for each PQ phenomenon (Table 15).

**Table 15:** CPQIs based on dynamic and static for different cases.

Scenario	Case 1	Case 2	Case 3	Case 4
	Non-Linear load	Unbalanced Load	Inductive load	Non-Linear, Un Balanced, and Inductive Loads
CPQI-Dynamic Weight ($\:{W}_{d}$)	1.425	1.500	0.748	1.593
CPQI-Static Weight ($\:{W}_{s}$)	0.450	0.734	0.385	0.949

**Fig. 14:**
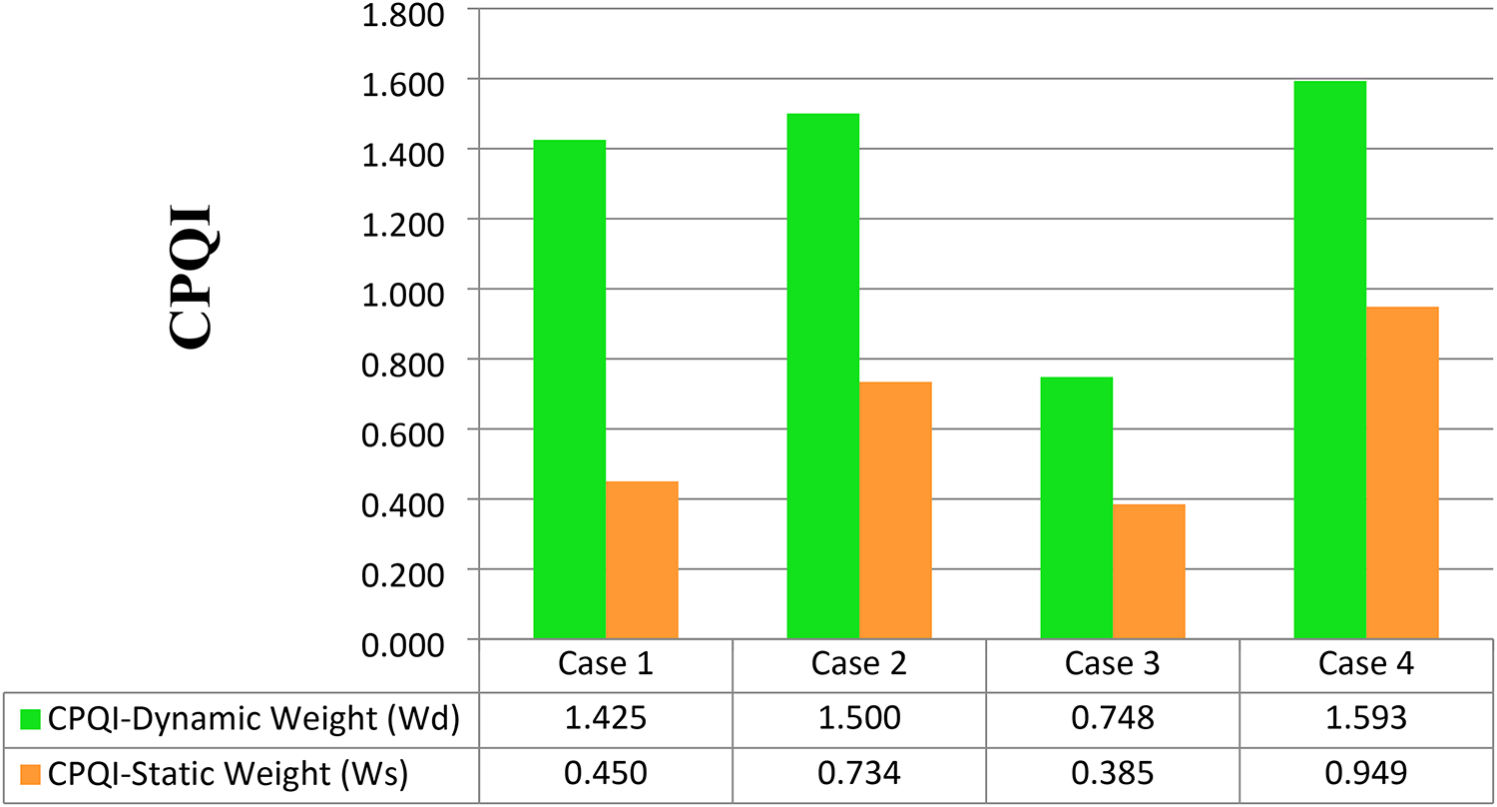
Comparative analysis of CPQIs under static and dynamic weights.

Figure 14 depicts the comparative analysis of the computed CPQI using static and dynamic weights. Based on the static weight, CPQIs under all cases are acceptable, although there are problems with some of the PQ phenomena under these abnormal conditions. On the other hand, by using the dynamic weight, the obtained CPQI values show that the overall PQ performance of the NRR network is not acceptable when a nonlinear load is added, as it is above unity. Additionally, when an unbalanced load is added, the results become worse than in the case of adding a nonlinear load. By adding the three abnormal conditions, which are nonlinear, unbalanced, and inductive loads, the CPQI of the system becomes worse than that in the previous two cases. The only acceptable case is when an inductive load is added. This means that using the dynamic weight can reflect the assessment more accurately.

## Conclusion

One of the key performance indicators for both future and contemporary power networks is the level of delivered power quality (PQ). Till now, there has been neither a common nor standardized way to describe and assess the overall PQ performance of the electrical network. This study proposed a model in MATLAB/Simulink to simulate a real electrical power system for an NRR and assess its PQ performance using multiple measures from a real facility and readings from the MATLAB Simulink model, taking into consideration the standard limit for each PQ index. Model verification and validation were performed under several normal and abnormal conditions, and the results confirmed the robustness and reliability of the simulated NRR mode. Several of the most important PQ indices namely; voltage and current imbalances, voltage and current harmonic distortions, and the power factor, are considered.

Since the decision problem of this study depends on several alternatives and multiple criteria, an MCDM methodology is used. The CRITIC method is an MCDM method that is used for obtaining objective weights, and compared with other objective weighting methods, this method has the advantages of strong operability and fewer calculations. Additionally, the AHP approach’s subjective weighting method is built based on experts’ opinions. This study presented a combined CRITIC-AHP approach for assessing overall PQ indices at an electrical network for a nuclear facility based on different PQ indices simultaneously. Objective weights for the different mentioned PQ phenomena of the NRR electrical system are determined using the measurements of these PQ phenomena. By analyzing the obtained weights and comparing them with the weights obtained from the subjective AHP methodology, it was found that the weights obtained from the two methodologies are close and compatible with the view of safety for this studied nuclear facility.

Finally, a new approach is proposed for computing the dynamic weights as a function of the static weights, the standard limits, and the measurements from the NRR MATLAB/Simulink model. Based on the obtained dynamic CRITIC-AHP weights, an overall power quality index was computed and analyzed to assess the PQ performance of this nuclear facility under different abnormal conditions. Under these assumed abnormal operating conditions, the combined power quality index of the proposed NRR facility was accepted when using static weights, which were determined by using the measurements under abnormal conditions but was not acceptable when using dynamic weights instead of static weights, which indicated that a dynamic weight is more accurate than a static weight for judging the overall system PQ. The obtained values of CPQI by applying the proposed approach in the test system under adding a nonlinear load, an unbalanced load, and an inductive load, and the three abnormal conditions together were 1.425, 1.5, 0.748, and 1.593, respectively, showing that the CPQI provides a reliable and comprehensive tool for monitoring the electrical network performance in nuclear facilities, ultimately contributing to their safe operation and grid stability.

Additional research can be performed to study other PQ phenomena and apply other abnormal operating conditions. Also, this proposed methodology can be applied when renewable energy sources are inserted into an electrical system to determine how this affects the PQ performance. Future work can also be extended by studying several PQ improvement techniques to enhance the combined PQ index and, consequently, the overall performance of the system. Additionally, this proposed technique can be applied to a real electrical network for assessing the overall power quality in real time.

## Data Availability

The datasets generated and/or analyzed during the current study are available from the corresponding author upon reasonable request.

## Abbreviations

AHP: Analytic Hierarchy Process
CB: Circuit Breaker
CRITIC: Criteria importance through intercriteria correlation
DBAs: Design Basis Accidents
DG1, DG2: Diesel Generator 1, 2
ESB-R: Essential Service Board- Right Busbar
ESB-L: Essential Service Board- Left Busbar
EW: Entropy Weight
LTS-L: Low Tension Switchboard- Left Busbar
LTS-R: Low Tension Switchboard- Right Busbar
MCDM: Multicriteria Decision-Making
NRR: Nuclear reactor research
PQ: Power Quality
PV: Photo Voltaic
Tr1, Tr2: Transformers 1, 2
TOPSIS: Technique for order preference by similarity to the Ideal Solution
UPS: Uninterruptible AC power supply

## Symbols

$$\:{\sigma\:}_{j}$$: Standard deviation
*CI*: Consistency index
*CR*: Consistency ratio
$\:{{F}_{m}}_{j}$: The current measurement value of PQ phenomena
$\:{{F}_{t}}_{j}$: Threshold (standard) limit value of PQ phenomena
$\:{I}_{h}$: The harmonic current
$\:{I}_{max}$: The phase current with maximum deviation from the average current (I_h_)
$\:{I}_{THD}$: Current harmonic distortion
$$\:{I}_{Un}$$: Current unbalance
*Lon*: Internal inductance
*n*: Number of compared elements (criteria)
*PF*: *PF* Power factor
*RI*: Random consistency index
*Ron*: Internal resistance
$\:{{S}_{m}}_{j}$: The score fr the measured jth PQ phenomenon
$\:{{S}_{t}}_{j}$: The score fr the threshold PQ phenomenon
$\:{V}_{ab},\:{V}_{bc},\:{V}_{ca}$: Three-line voltages
$\:{V}_{abe}$: The difference between the line voltage and the average line voltage
$\:{v}_{h}$: The harmonic voltage
$\:{W}_{a}$: The weight of each criterion by AHP
$\:{W}_{c}$: Objective weight by CRITIC
$$\:{W}_{s}$$: Static weight
$$\:{W}_{d}$$: Dynamic weight
$\:{\left[{x}_{ij}\right]}_{m\text{*}n}$: This decision matrix for *m* alternatives and *n* criteria
$\:\stackrel{-}{{x}_{ij}}$: The normalized decision matrix
$\:{x}_{ij}$: The actual value of alternative i for criterion *j*
$\:{x}_{j}^{worst}$: The criterion worst value
$\:{x}_{j}^{best}$: The criterion best value

## References

[1] Singh, U. A Research Review on Detection and Classification of Power Quality Disturbances caused by Integration of Renewable Energy Sources, [Online]. (2020). Available: https://www.researchgate.net/publication/344373409

[2] Devaraju, T. & Power Quality Course Material Accessed: Feb. 25, 2024. [Online]. (2020). Available: https://www.svec.education/wp-content/uploads/2020/01/power-quality-course-material.pdf

[3] IEEE Power and Energy Society. Transmission and distribution Committee, 1159–2019 - IEEE Recommended Practice for Monitoring Electric Power Quality. IEEE, (2019).

[4] Kedarnath Magadum, P. & Sakri, S. G. Effects of Non Linear Loading on Power Quality, [Online]. (2023). Available: https://www.researchgate.net/publication/372139118

[5] Dhote, P. V., Deshmukh, B. T. & Kushare, B. E. *Generation of Power Quality Disturbances Using MATLAB-Simulink*. (2015).

[6] Nassar, S. R., Eisa, A. A., Saleh, A. A., Farahat, M. A. & Abdel-Gawad, A. F. Evaluating the Impact of Connected Non Linear Loads on Power Quality- a Nuclear Reactor case study, *J Radiat Res Appl Sci*, vol. 13, no. 1, pp. 688–697, Jan. (2020). 10.1080/16878507.2020.1828018

[7] Kamarposhti, M. A., Colak, I., Thounthong, P. & Eguchi, K. Modeling and Simulation of DVR and D-STATCOM in Presence of wind Energy System. *Computers Mater. Continua*. **74** (2), 4547–4570. 10.32604/cmc.2023.034082 (2023).

[8] Attar, H., Kamarposhti, M. A. & Solyman, A. A. A. Impacts of integration of wind farms on voltage stability margin. *Int. J. Electr. Comput. Eng.* **12** (5), 4623–4631. 10.11591/ijece.v12i5.pp4623-4631 (Oct. 2022).

[9] Srilakshmi, K. et al. Development of renewable energy fed three-level hybrid active filter for EV charging station load using Jaya grey wolf optimization. *Sci. Rep.* **14** (1). 10.1038/s41598-024-54550-7 (Dec. 2024). 10.1038/s41598-024-54550-7 PMC11310476 38396163

[10] Shokouhandeh, H., Ahmadi Kamarposhti, M., Asghari, F., Colak, I. & Eguchi, K. Distributed Generation Management in Smart Grid with the Participation of Electric Vehicles with respect to the Vehicle Owners’ opinion by using the Imperialist competitive algorithm. *Sustain. (Switzerland)*. **14** (8). 10.3390/su14084770 (Apr. 2022).

[11] Kamarposhti, M. A. & Lesani, H. *Comparison between Parallels and Series FACTS Devices on Static Voltage Stability Using MLP Index* (IEEE, 2010).

[12] Kamarposhti, M. A. & Lesani, H. Effects of STATCOM, TCSC, SSSC and UPFC on static voltage stability, *Electrical Engineering*, vol. 93, no. 1, pp. 33–42, Mar. (2011). 10.1007/s00202-010-0187-x

[13] Shokouhandeh, H., Kamarposhti, M. A., Colak, I. & Eguchi, K. Unit commitment for power generation systems based on prices in smart grid environment considering uncertainty, *Sustainability (Switzerland)*, vol. 13, no. 18, Sep. (2021). 10.3390/su131810219

[14] Mojtahedzadeh Larijani, M., Ahmadi Kamarposhti, M. & Nouri, T. Stochastic Unit Commitment Study in a Power System with Flexible Load in Presence of High Penetration Renewable Farms, *Int J Energy Res*, vol. 2023, (2023). 10.1155/2023/9979610

[15] Makahleh, F. M. et al. Optimal Management of Energy Storage Systems for Peak shaving in a Smart Grid. *Computers Mater. Continua*. **75** (2), 3317–3337. 10.32604/cmc.2023.035690 (2023).

[16] Habib, S., Ahmadi Kamarposhti, M., Shokouhandeh, H., Colak, I. & Barhoumi, E. M. Economic dispatch optimization considering operation cost and environmental constraints using the HBMO method, *Energy Reports*, vol. 10, pp. 1718–1725, Nov. (2023). 10.1016/j.egyr.2023.08.032

[17] Kamarposhti, M. A. et al. Optimum operation management of microgrids with cost and environment pollution reduction approach considering uncertainty using multi-objective NSGAII algorithm. *IET Renew. Power Gener.*10.1049/rpg2.12579 (2022).

[18] Srilakshmi, K. et al. Design of solar and energy storage systems fed reduced switch multilevel converter with flower pollination optimization, *J Energy Storage*, vol. 99, Oct. (2024). 10.1016/j.est.2024.113324

[19] Srilakshmi, K. et al. Optimal design of hybrid green energy powered reduced switch converter based shunt active power filter using horse herd algorithm. *Sci. Rep.* **14** (1). 10.1038/s41598-024-71100-3 (Dec. 2024). 10.1038/s41598-024-71100-3 PMC11372080 39227381

[20] Srilakshmi, K. et al. A New Control Scheme for Wind/Battery Fed UPQC for the Power Quality Enhancement: a hybrid technique. *IETE J. Res.*10.1080/03772063.2024.2370959 (2024).

[21] Aljafari, B., Alapati, Y. K., Srilakshmi, K., Balachandran, P. K. & Thanikanti, S. B. An optimized neural network-honey badger based control technique for a hybrid solar PV and battery energy storage fed unified power quality conditioner, *J Energy Storage*, vol. 106, Jan. (2025). 10.1016/j.est.2024.114818

[22] Singh, A., Kumar Malik, S. & Review Major MCDM Techniques and their application-A (2014). [Online]. Available: www.iosrjen.org.

[23] Soniya, S., Ramachandran, M., Sathiyaraj, C. & Mathivanan, G. A Review on Multi-Criteria Decision-Making and Its Application, *REST Journal on Emerging trends in Modelling and Manufacturing*, vol. 7, no. 4, pp. 101–107, Dec. (2021). 10.46632/7/4/1

[24] Elsotohy, A. M., Soliman, A. M. A., Adail, A. S., Eisa, A. A. & Othman, E. Comprehensive power quality performance assessment for electrical system of a nuclear research reactor, *Sci Rep*, vol. 13, no. 1, Dec. (2023). 10.1038/s41598-023-36692-2 10.1038/s41598-023-36692-2 PMC10279760 37337023

[25] Sahoo, S. K. & Shubhra Goswami, S. A Comprehensive Review of multiple criteria decision-making (MCDM) methods: advancements, applications, and future directions. *Decis. Mak. Adv. J. Homepage: Www dma-journalorg*. **1** (1), 25–48. 10.31181/dma1120237 (2023).

[26] Parvaneh, F. & Hammad, A. Application of Multi-criteria decision-making (MCDM) to select the most sustainable Power-Generating Technology. *Sustain. (Switzerland)*. **16** (8). 10.3390/su16083287 (Apr. 2024).

[27] Amusan, O. T., Nwulu, N. I. & Gbadamosi, S. L. Multi-criteria decision-based hybrid energy selection system using CRITIC weighted CODAS approach. *Sci. Afr.* **26**10.1016/j.sciaf.2024.e02372 (Dec. 2024).

[28] Sahoo, S. K. & Goswami, S. S. A Comprehensive Review of Multiple Criteria Decision-Making (MCDM) Methods: Advancements, Applications, and Future Directions, *Decision Making Advances*, vol. 1, no. 1, pp. 25–48, Dec. (2023). 10.31181/dma1120237

[29] Worku, T. T. Multi-steps integrated mathematical MCDM model for construction material selection of Addis Ababa high-rise building infrastructure. *Urban Plan. Transp. Res.* **12** (1). 10.1080/21650020.2024.2329201 (2024).

[30] Bohra, S. S. & Anvari-Moghaddam, A. A comprehensive review on applications of multicriteria decision-making methods in power and energy systems, Mar. 25, *John Wiley and Sons Ltd*. (2022). 10.1002/er.7517

[31] Bajaj, M. & Singh, A. K. An analytic hierarchy process-based novel approach for benchmarking the power quality performance of grid-integrated renewable energy systems, *Electrical Engineering*, vol. 102, no. 3, pp. 1153–1173, Sep. (2020). 10.1007/s00202-020-00938-3

[32] Bajaj, M. et al. Power Quality Assessment of distorted distribution networks incorporating renewable distributed Generation systems based on the Analytic Hierarchy process. *IEEE Access.* **8**, 145713–145737. 10.1109/ACCESS.2020.3014288 (2020).

[33] Bajaj, M. & Singh, A. K. A global power quality index for assessment in distributed energy systems connected to a harmonically polluted network. *Energy Sources Part. A: Recovery Utilization Environ. Eff.*10.1080/15567036.2021.1929577 (2021).

[34] Chen, Y. A Novel Approach for Evaluating Power Quality in Distributed Power Distribution Networks Using AHP and S-Transform, *Energies (Basel)*, vol. 17, no. 2, Jan. (2024). 10.3390/en17020411

[35] Thirumala, K., Budhavarapu, J. & Devara, H. B. A composite power quality index for low-voltage active distribution networks, *International Journal of Emerging Electric Power Systems*, vol. 22, no. 3, pp. 339–351, Jun. (2021). 10.1515/ijeeps-2020-0225

[36] Shi, H., Li, Y., Jiang, Z. & Zhang, J. Comprehensive power quality evaluation method of microgrid with dynamic weighting based on CRITIC. *Meas. Control (United Kingdom)*. **54**, 5–6. 10.1177/00202940211016092 (May 2021).

[37] Sacasqui, M., Luyo, J. & Delgado, A. *A Unified Index for Power Quality Assessment in Distributed Generation Systems using Grey Clustering and Entropy Weigh*. (2018).

[38] Yuan, X. et al. Comprehensive quality evaluation method of smart meters based on AHP-Critic, in *Journal of Physics: Conference Series*, IOP Publishing Ltd, May (2021). 10.1088/1742-6596/1920/1/012010

[39] Zhao, Y., Li, P., Wang, T., Kang, Y. & Zhao, Y. B. Equipment Health Assessment Based on AHP-CRITIC Dynamic Weight, in *Chinese Control Conference, CCC*, IEEE Computer Society, pp. 5841–5846. (2022). 10.23919/CCC55666.2022.9902488

[40] Jiang, Y., Fang, M., Liu, Z. & Wang, W. Comprehensive evaluation of power quality based on an improved TOPSIS method considering the correlation between indices. *Appl. Sci. (Switzerland)*. **9** (17). 10.3390/app9173603 (Sep. 2019).

[41] Ji, Y. et al. Fuzzy evaluation method of power quality based on improved CRITIC and AHP, in *7th International Conference on Information, Cybernetics, and Computational Social Systems, ICCSS 2020*, Institute of Electrical and Electronics Engineers Inc., Nov. 2020, pp. 635–639. (2020). 10.1109/ICCSS52145.2020.9336841

[42] Saaty, T. L. & DECISION MAKING-THE ANALYTIC HIERARCHY AND NETWORK PROCESSES (AHP/ANP), (2004).

[43] Nyimbili, P. H. & Erden, T. A Hybrid Approach Integrating Entropy-AHP and GIS for Suitability Assessment of Urban Emergency Facilities, *ISPRS Int J Geoinf*, vol. 9, no. 7, Jul. (2020). 10.3390/ijgi9070419

[44] Bhattacharyya, S. & Cobben, S. Consequences of poor power Quality-An overview, (2011). [Online]. Available: www.intechopen.com.

[45] Markiewicz, H. & Klajn, A. Power Quality Application Guide, [Online]. (1999). Available: www.lpqi.org

[46] Lee, B. & Kim, K. M. *Unified Power Quality Index Based on Value-Based Methodology* (IEEE, 2009).

[47] IAEA-TECDOC-1625. Accessed: Feb. 25, 2024. [Online]. Research Reactor Modernization and Refurbishment, (2009). Available: https://www-pub.iaea.org/MTCD/Publications/PDF/te_1625_web.

[48] Mahmoud, M. R., Amin, E. A., Zaky, M. M., Abdelsalam, A. & Bahr, W. Calculation of dose rates in loss of coolant accident due to double ended rupture of the experimental tangential irradiation beam tube of MTR reactor., *Arab Journal of Nuclear Sciences and Applications*, vol. 0, no. 0, pp. 0–0, Nov. (2021). 10.21608/ajnsa.2021.75275.1472

[49] Altaira, M. & Issa, A. Voltage unbalance impact on the characteristics of three phase induction motor using Matlab/Simulink. *Int. J. Sci. Stud. Publishing*. **11** (3), 9–21 (2021).

[50] Nassar, S. M., Eisa, A. A., Saleh, A. A., Farahat, M. A. & Abdel-Gawad, A. F. Studying the Influence of Connecting New Energy Saving Loads on the Electrical Power System of Nuclear Facility, [Online]. (2021). Available: https://eijest.journals.ekb.eg/

[51] IEEE Industry Applications Society. *Power Systems Engineering Committee., IEEE Recommended Practice for Electric Power Distribution for Industrial Plants* (Institute of Electrical and Electronics Engineers, 1994).

[52] IEEE Power and Energy Society, “IEEE Recommended Practice and Requirements for Harmonic Control in Electric Power Systems, IEEE Std 519-2014 (Revision of IEEE Std 519-1992).” Available: https://ieeexplore.ieee.org/document/6826459.

[53] B. Hussein Mohamed El-eissawi Fathi and M. N. Zaher Ayad G Abdel Salam, “Power Quality Assessment,” 2012. Available: https://inis.iaea.org/records/kbjwg-2as06/files/45024023.

[54] Zaher, M., Ayad, N., Elsherbiny, E., Abdelsalam, G. & Eleissawi, H. “Investigation And Mitigation Techniques Of Power Quality Problems In Nuclear Installations,” 2013. Available: https://www.academia.edu/93979937.

